# Structure–transport relationships in MXene-based membranes: from synthesis to selective water treatment

**DOI:** 10.1039/d6na00225k

**Published:** 2026-09-01

**Authors:** Salman Khan, Tanvir Khan, Muhammad Naveed, Salman Khan, Ajmal Shah, Naveed Ahmad, Shohreh Azizi, Malik Maaza

**Affiliations:** a Department of Chemistry, Abdul Wali Khan University Mardan Mardan 23200 Pakistan; b School of Chemistry, Xian Jiaotong University 710049 Xian people's Republic of China; c UNESCO-UNISA Africa Chair in Nanosciences/Nanotechnology, College of Graduate Studies, University of South Africa Muckleneuk Ridge Pretoria 0181 South Africa azizis@unisa.ac.za shohrehazizi1379@gmail.com; d Department of Chemical and Materials Engineering, College of Engineering, Northern Border University Arar Saudi Arabia

## Abstract

Two-dimensional laminar membranes with precisely controlled nanochannels have attracted significant attention for overcoming the permeability selectivity trade-off in water purification. Among them, MXene-based membranes exhibit unique advantages arising from their hydrophilic surfaces, rich surface terminations, and tunable interlayer spacing. This review presents MXene synthesis routes, including direct and *in situ* etching, hydrothermal processing, and emerging green approaches, with emphasis on their influence on flake morphology, surface chemistry, and stacking behavior. Membrane fabrication strategies, such as vacuum-assisted filtration, mixed-matrix integration, and interlayer engineering, are analyzed to establish correlations between structural features and separation performance. Particular attention is given to transport mechanisms within MXene nanochannels, where size sieving, electrostatic interactions, and confined transport collectively govern ion and molecule selectivity. Recent advances in crosslinking and nanoparticle intercalation are highlighted for effectively suppressing swelling and stabilizing angstrom-scale channels under aqueous conditions. MXene membranes demonstrate high rejection efficiencies with enhanced water permeance and antifouling properties across desalination, heavy metal removal, and organic contaminant separation. The remaining challenges, including scalable fabrication, structural stability, and defect control, are discussed to guide future development toward practical membrane applications.

## Abstract 2

Two-dimensional laminar membranes with precisely controlled nanochannels have attracted significant attention for overcoming the permeability selectivity trade-off in water purification.

## Graphical abstract

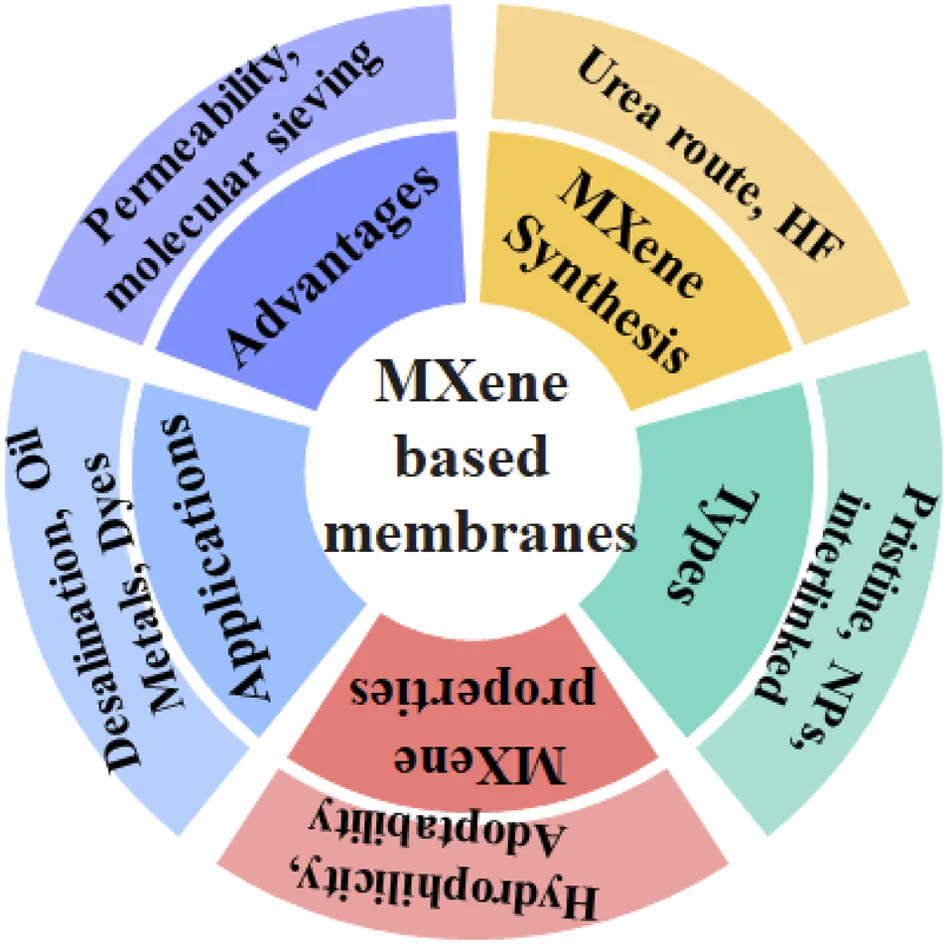

## 1 Introduction

Natural and man-made water pollution has become an important worldwide issue with both short-term and long-term consequences on the health of humans and the ecological balance.^1,2^ Although water covers more than 70% of the Earth's surface,^3^ the availability of safe drinking water remains limited. Only 0.002% of the world's water resources are accessible for human consumption, and consequently, waterborne infections account for nearly 80% of global diseases according to the World Health Organization (WHO).^4,5^ The WHO further estimated that one-sixth of the world population (1.1 billion) is still affected by the lack of safe drinking water.^6,7^ The threat is enhanced by the presence of chemical contaminants, such as dyes, pharmaceuticals, heavy metals, pesticides, and petrochemicals, which have harsh toxic effects on aquatic organisms and, in turn, on human health by bioaccumulation.^4,8^ Among these contaminants, particular attention has been given to heavy metal (HM) pollution because of their persistence, non-biodegradability, and high toxicity.^9^ Combustion of coal, electroplating, mining, and leather tanning are industrial and urban operations that contribute significantly to HM pollution in aquatic systems.^10^ Many studies have been cited with increasing levels of metals in rivers, whereby the levels of Cr, As, Cd, Cu, Pb, and Zn are 7.7 µg L^−1^, 1.71 µg L^−1^, 16.0 µg L^−1^, 96.8 µg L^−1^, 7.5 µg L^−1^, and 30.1 µg L^−1^, respectively.^11^ The high values are usually linked to industrial effluents, agricultural runoffs, and poor waste management that cumulatively increase the level of heavy metal pollution in lower stream regions of rivers.^12^

The global data set, comprising more than 15 million monthly records of electrical conductivity of surface waters collected between 1980 and 2023, indicates that the global median electrical conductivity of surface waters is approximately 509–205 µS cm^−1^, with nearly 60% of rivers falling in the range of 50 to 500 µS cm^−1^^13^ This has been significantly enhanced by human activities, especially through industrial discharge and agricultural runoff. As an example, there is sugarcane vinasse, which is a typical agro-industrial waste, which can reach salinity rates of 8420 µS cm^−1^ and sodium levels of about 60 mg L^−1^, demonstrating the scale of anthropogenic factors in freshwater salinization on a global scale.^13^ Moreover, the effect of organic pollutants in the form of untreated sewage, industrial processes, and animal agriculture is still worsening the situation of water quality in the world. These pollutants greatly increase the biological oxygen demand (BOD) of rivers, lowering dissolved oxygen ratios necessary for aquatic organisms.^14^ In 2000, approximately 1.1 billion individuals (19% of the world population) resided in the vicinity of rivers that had BOD levels greater than 5 mg L^−1^, and it is estimated that by 2050, the population will grow to 2.5 billion (26%) due to increased pressure of pollution.^15^ The constant release of massive amounts of organic contaminants, both in single-compound and mixed forms, by point and non-point sources, has continued to damage freshwater quality across the globe.^15–17^ In a comprehensive global meta-analysis of 311 studies, 5315 samples, and 56 countries conducted between 1970 and 2020, *p*,*p*′-DDT was found to have the highest loadings, especially in the source of drinking water.^14,18^ In addition to this, Monte Carlo risk simulations indicated 8.22 × 10^−7^ carcinogenic risks in children and 3.83 × 10^−7^ in adults, with 75% of the simulated child exposures and 95% of the simulated adult exposures falling below 1.07 × 10^−6^ and 7.35 × 10^−6^ thresholds, respectively, of established international risk assessment values.^18^

Contamination of water is prevalent, including not only dyes and salts, but also organic contaminants and persistent organic compounds.^19^ To address these threats, various wastewater treatment and water purification technologies have since been developed to reduce the threat and enhance the quality of effluent before discharge or reuse.^20^ Chemical techniques, such as coagulation, flocculation, and advanced oxidation processes (AOPs) (*e.g.*, Fenton, photo-catalysis, ozone, and electro-oxidation) are very common in the degradation of recalcitrant organic pollutants.^21^ Biological treatment, which includes activated sludge, membrane bioreactors, and sequencing batch reactors, further decreases the organic load and BOD using microbial activity.^22^ Chemical methods, including coagulation, flocculation, and AOPs (*e.g.*, Fenton, photo-catalysis, ozone, and electro-oxidation), are widely used to degrade recalcitrant organic pollutants. Biological treatments, such as activated sludge systems, membrane bioreactors, and sequencing batch reactors, further reduce organic load and BOD through microbial activity.^23^ More developed hybrid systems, combining various technologies, have greater pollutant removal efficiencies and would be sustainable objectives in water reuse and zero liquid discharge.^24^

**Fig. 1:**
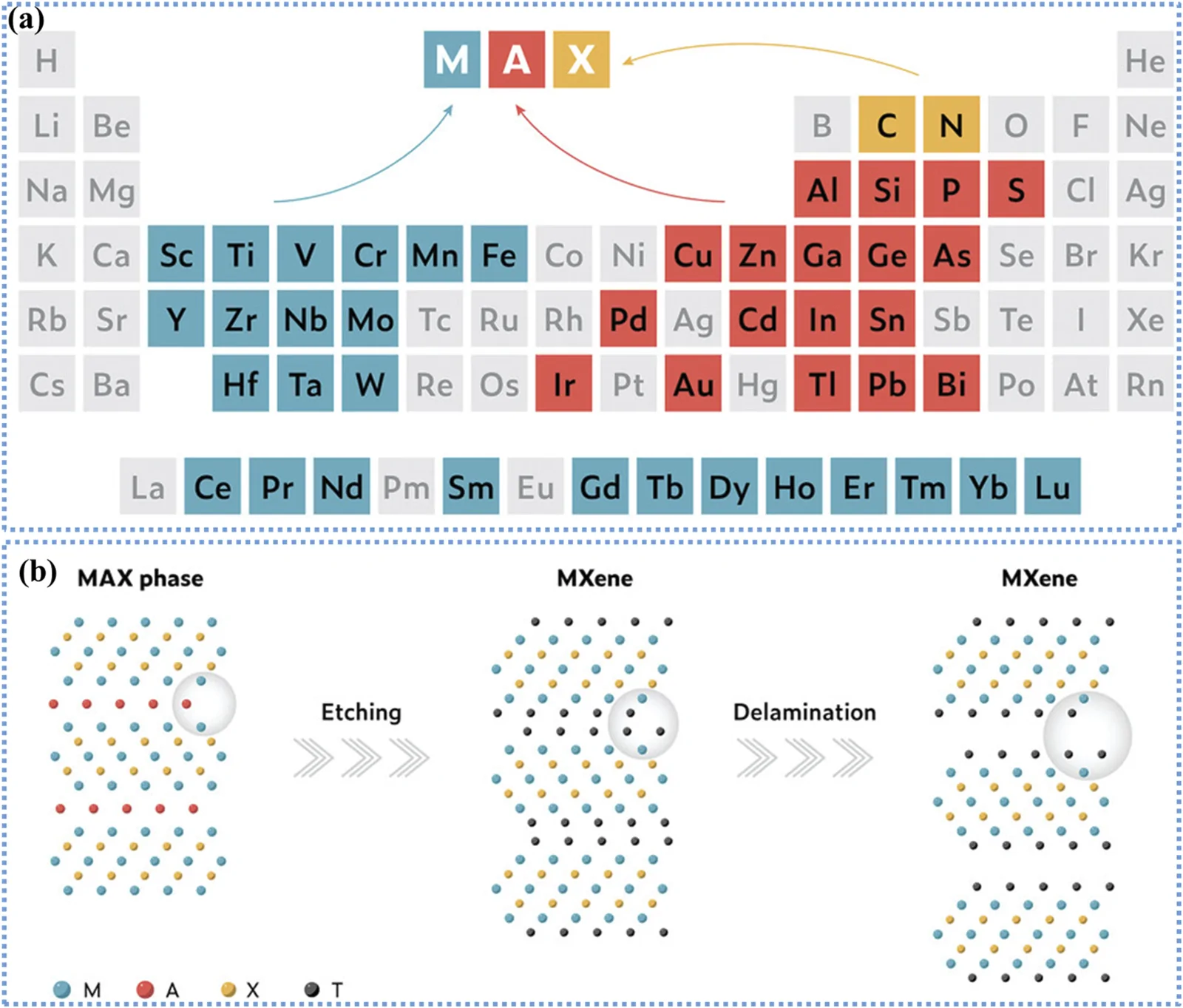
(a) The periodic table of elements, where the “M” “A” and “X” elements of the known MAX phases are highlighted. (b) Schematics of the synthesis process of MXenes from MAX phases. Here M_3_AX_2_ MAX phase and M_3_X_2_T_*x*_ MXenes are shown as examples. Reproduced with permission from ref. 51. Copyright 2022, Jhon Wiley and Sons.

The past few years have seen impressive innovations in membrane technologies driven by the increasing need to develop separation systems with higher efficiency, selectivity, mechanical stability, and long-term operational stability in various water treatment and industrial processes.^25^ Membrane-based processes have now become essential in recent separation methods, especially in water purification, where microfiltration (MF), ultrafiltration (UF), nano-filtration (NF), reverse osmosis (RO), and modified polyvinylidene fluoride (PVDF) membranes have been embraced due to their proven effectiveness.^26–30^ However, high-performance polymeric membranes, such as thin-film composites and surface-modified nanocomposites, remain susceptible to severe fouling (organic, inorganic, and biofouling) and structural degradation over prolonged operation. Such issues inevitably decrease the permeate flux, increase cleaning frequency and energy consumption, and reduce membrane life hence, there is a need to develop more robust substitutes.^31,32^ Consequently, a wide range of next-generation membranes, including nanocomposite membranes, mixed-matrix membranes (MMMs), and graphene and MXene-based membranes, have been produced to address the inherent drawbacks of traditional polymeric membranes.^33,34^ These novel materials incorporate functional nanomaterials and engineered surface chemistries that significantly improve permeability, mechanical and chemical resistance, and pollutant rejection, and overcome chronic problems, including membrane fouling, low selectivity, and limited operation ranges.^35^ The MXene-based membranes, specifically those based on Ti_3_C_2_T_*x*_, have proven to be highly promising because of their high hydrophilicity, tunable interlayer spacing, controllable surface chemistry (Fig. 1a and b), and a unique combination of charge and size-selective transport properties.^36^ Unlike graphene oxide (GO) membranes, MXene laminates enhance rejection of large ions and dyes while maintaining rapid transport pathways for single-charged ions. Their negatively charged, conductive, and customizable nanochannels enable efficient separation of multivalent toxic metal ions such as Pb^2+^, Ni^2+^, and Al^3+^, making them particularly effective for desalination, water softening, and heavy-metal remediation.^37^

One of the main strengths of the MXene materials is a high specific surface area (>1000 m^2^ g^−1^), due to their 2D layered structure, delamination, and the presence of abundant surface functional groups.^38^ This high surface area provides many active adsorption sites and a strong interaction with pollutants, enabling the rapid and effective removal of a large number of contaminants. Many different MXene-based materials have exhibited excellent adsorption capabilities, such as a Pb^2+^ uptake of up to 382.7 mg g^−1^ in MXene–alginate composites, and fast Cu^2+^ removal (87.6 mg g^−1^).^39^ Hybrid materials like MoS_2_–MXene aerogels demonstrate extremely high adsorption capacities up to approximately 932.8 mg g^−1^ of Hg^2+^. On the same note, functionalized MXenes show very high performance in adsorption, with values of more than 4800 mg g^−1^^40^ The net effect of these results is that the structural tuneability and the high surface area of MXenes correlate directly with better water purification performance.^41^ Alongside the high adsorption potential, MXene-based membranes are highly hydrophilic because of the presence of –OH, –O, and –F surface functional groups that improve rapid water transport and minimize fouling.^42–44^ For instance, the MXene/PES composite membrane exhibits a contact angle of 0°, meaning that water droplets spread instantly across the membrane surface, resulting in high water flux along with stable dye and salt rejection.^42,43^ Water permeance is further increased by tunable hydrophilicity in polymer functionalization or material mixing, which also increases the desalination efficiency.^42,43^ The other essential functional characteristic of MXene membranes is the adjustable interlayer spacing, which determines the molecular transport and separation efficiency.^45^ Although narrow channel spacing can hinder diffusion, the addition of intercalating agents like Si-based species increases the *d*-spacing to approximately 11 Å, which improves sheet alignment, reduces structural defects, and significantly enhances both permeability and separation performance. These adjustments have allowed MXene membranes to reach up to 99% of separation efficiency with stable flux when using oily wastewater.^46^ In addition to increased permeability and selectivity, MXene membranes exhibit excellent antibacterial activity due to their sharp sheet edges, reactive surface defects, and oxidative activity, all of which destabilize bacterial cell membranes and prevent the growth of Gram-positive and Gram-negative species.^47^ The addition of Ag or ZnO materials further enhances sterilization functionality with Ag or ZnO-containing composites providing close to 100% bacterial inhibition and greatly reducing biofilm formation, aiding in the long-term stability of operation in water purification systems.^48^ Furthermore, surface functionalization methods, including amino acid modification or intercalation using ammonium acetate and 2-alpha-Co(OH)_2_ nanosheets, promote the creation of hydration layers and distributions of surface charges, which result in high antifouling and a flux recovery ratio of about 98%.^49,50^

This review provides a comprehensive and systematic discussion of several important aspects of MXene membranes. The focus of this review is to provide a detailed description of the synthesis procedures of MXenes, such as hydrothermal methods, *in situ* HF etching, and the urea-glass approach, which provide a fluoride-free and scalable method to produce MXenes without the production of toxic waste. It also summarizes the fabrication and modification strategies of membranes and the categorization of MXene membranes. Moreover, this review presents a clear correlation between the chemistry of the cross-links (covalent, ionic, and organic pillaring) and the suppression of aqueous swelling, with a direct correlation between the interlayer spacing and its stabilization with an accuracy of a few Angstroms, thus breaking the permeability-selectivity trade-off. With the support of MD simulations, the coupled transport mechanisms in MXene nanochannels, namely size sieving, electrostatic Donnan exclusion, and confined water dynamics, are systematically explored, and how the surface terminations (–O, –OH, and –F) control the degree of ion dehydration and water friction. The rejection efficiency of MXene membranes against different types of water contaminants such as salts, oil–water emulsions, dyes, heavy metals, and bacterial inhibition is then critically analyzed with respect to surface functional groups, interlayer spacing, and nanochannel architecture. The structure–transport relationship of MXene membranes is discussed to enhance the water purification performance. Additionally, new progress in multifunctional hybrid MXene membranes with CNTs, MOFs, ZnO, and Ag nanoparticles is discussed, where synergies for high flux separation, self-cleaning under UV-irradiation, and antibacterial activity of well over 99% are demonstrated. Finally, this contribution offers a critical evaluation of the long-term operational stability, mechanical robustness under hydraulic stress, and real-world anti-fouling performance, which fills a gap between laboratory success and real-world water purification applications.

## 2 Synthesis and properties of MXenes and their membranes

Simultaneous with their increasing uses, considerable advances have been achieved in the synthesis of MXene precursors and over 70 MAX phases have been synthesized successfully to offer a versatile compositional platform upon which MXenes may be built. The MXene family has expanded rapidly and currently includes representative materials such as Ti_2_C, Ti_3_C_2_, (Ti_1_/_2_ Nb_1_/_2_)_2_C, (V_1_/_2_Cr_1_/_2_)_3_C_2_, Nb_2_C, Ti_3_CN, Ta_4_C_3_, V_2_C, and Nb_4_C_3_.^52^ Ti_3_C_2_T_*x*_ has so far become the most widely studied among the different MXene compositions because of its great selectivity and strong adsorption affinity to harmful polluters in water and wastewater systems.^53^ Notably, Ti_3_C_2_T_*x*_ was the first MXene to be synthesized and remains the most widely studied owing to its relatively facile synthesis, favorable mechanical robustness, and superior physicochemical performance.^54^

### 2.1. Synthesis of MXenes

#### 2.1.1. Direct hydrofluoric acid etching

**Fig. 2:**
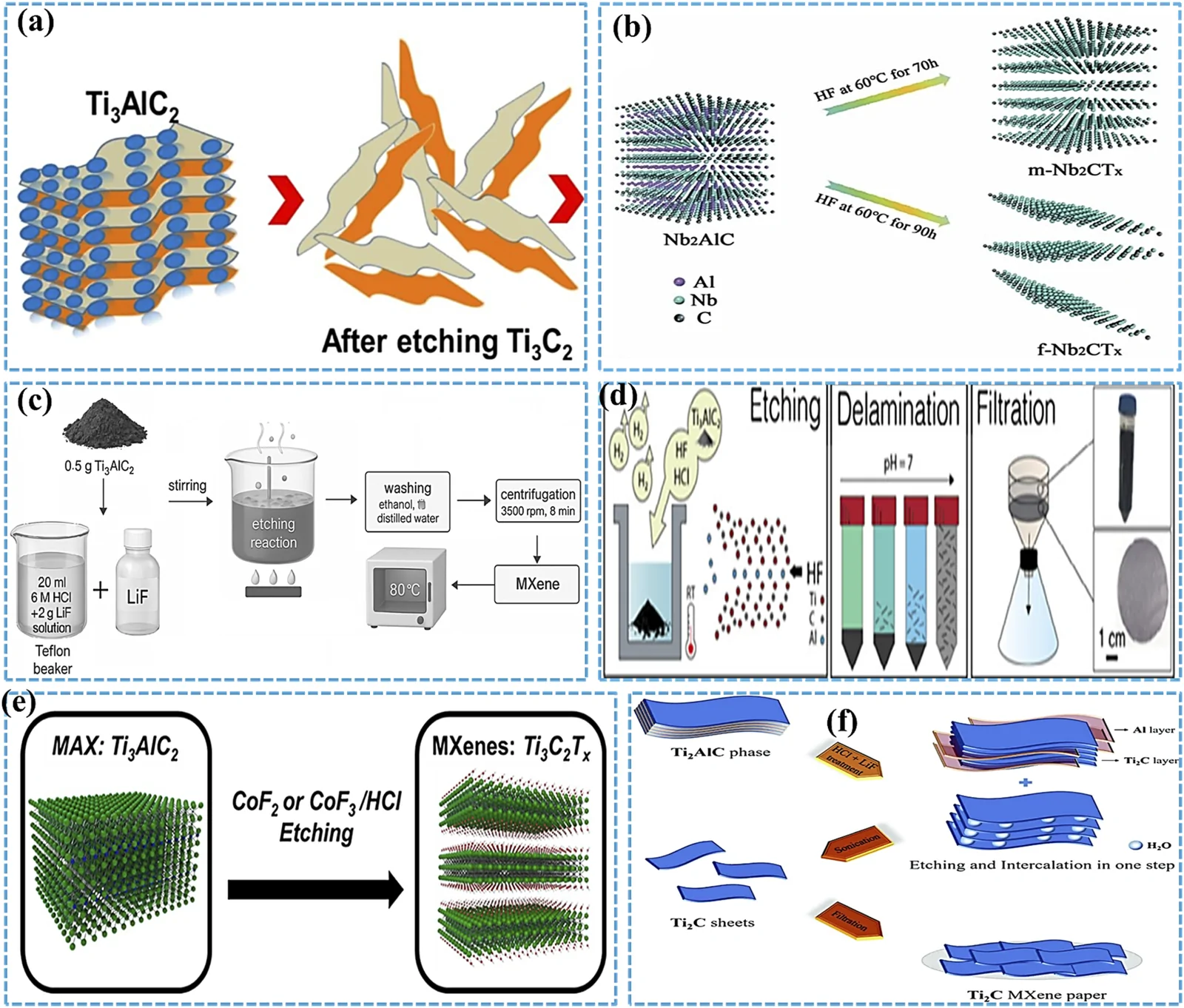
(a) Schematic diagram of the Ti_3_C_2_ MXene synthesis method. Reproduced with permission from ref. 56. Copyright 2024, Elsevier. (b) Model diagrams of the synthesis of accordion-like m-Nb_2_CT_*x*_ and f-Nb_2_CT_*x*_ nanosheets. Reproduced with permission from ref. 57. Copyright 2019, Royal Society of Chemistry. (c) Synthesis of Ti_3_C_2_T_*x*_ by direct HF etching. Reproduced from Springer Nature from ref. 58, which is open access and permits unrestricted use of materials under the terms of Creative Commons CC-BY. (d) Schematic of MXene synthesis. Reproduced with permission from ref. 59. Copyright 2023, Taylor and Francis. (e) Etching MAX using CoF_2_ or CoF_3_/HCl solution to synthesize MXene phases; reproduced with permission from ref. 66. Copyright 2019, American Chemical Society. (f) Schematic illustration of the MXene nanosheet preparation. Reproduced from Springer Nature from ref. 67, which is open access and permits unrestricted use of materials under the terms of Creative Commons CC-BY.

One of the earliest and most widely adopted approaches for MXene synthesis is the direct hydrofluoric acid etching of MAX phase precursors, particularly for the preparation of Ti_3_C_2_T_*x*_. In this method, the MAX phase is immersed in HF solution, where the selective removal of the A layer (typically Al) occurs, resulting in the formation of multilayered MXene powders. Subsequently, hydrogen bonding and van der Waals interactions yield accordion-like MXene structures (Fig. 2a).^55,56^ The chemical reactions involved in the HF etching process can be described using reactions (1) and (2):

1 M_*n*+1_AX_*n*_ + 3HF → M_*n*+1_X_*n*(s)_ + AF_3_ + 1.5H_2(g)_

2 M_*n*+1_X_*n*(s)_ + 2HF → M_*n*+1_X_*n*_F_2_ + H_2(g)_

For instance, few-layered Nb_2_CT_*x*_ (f-Nb_2_CT_*x*_) nanosheets have been synthesized by etching 0.5 g of Nb_2_AlC powder with 40% HF at 60 °C under continuous stirring for 90 h, while accordion-like multilayered Nb_2_CT_*x*_ structures were obtained under similar conditions, however, with a reduced etching duration of 70 h, as illustrated in Fig. 2b.^57^ Several studies have further optimized the direct HF route for Ti_3_C_2_T_*x*_ synthesis. For instance, Kiran *et al.* reported a controlled etching process in which Ti_3_AlC_2_ powder was gradually added to 5% HF under continuous stirring. Because the reaction is strongly exothermic and accompanied by gas evolution, slow addition of the MAX phase was necessary to maintain reaction stability. The mixture was stirred at 50 °C for 24 h, followed by repeated washing and centrifugation until neutral pH was achieved. The final product was dried under vacuum to obtain layered MXene powders (Fig. 2c).^58^ In a similar manner, Shuck *et al.* described the synthesis routes of both small-scale and large-scale synthesis in mixed acidic systems that contained HF, HCl, and water (Fig. 2d). Ti_3_AlC_2_ was etched by the small-batch process at 35 °C with controlled stirring for 24 h, whereas a specially designed reactor provided gram-scale production without depleting temperature using a cooling jacket system. The article demonstrated the possibility of scaling up the direct HF procedure to produce MXenes on a large scale.^59^ Wang *et al.* conducted another experiment to determine the effect of the etching time by varying the reaction time between several hours and 17 days with the use of 40% HF at room temperature. The resulting multilayer Ti_3_C_2_T_*x*_ particles were washed by centrifugation, vacuum dried, and finally transformed into electrode materials to ensure that long-term etching influences structural expansion and surface termination density.^60^ Naguib *et al.* reported that two-dimensional Ti_3_C_2_ MXene nanocrystals can be prepared by reacting Ti_3_AlC_2_ powders with 50% HF at room temperature in the course of about 2 h.^61,62^ The reaction equations of this process are as follows:

3 Ti_3_AlC_2_ + 3HF → Ti_3_C_2_ + AlF_3_ + 3/2H_2_

4 Ti_3_C_2_ + 2H_2_O → Ti_3_C_2_(OH)_2_ + H_2_

5 Ti_3_C_2_ + 2HF → Ti_3_C_2_F_2_ + H_2_

Reaction (3) is the primary etching step, followed by surface functionalization reactions (4) and (5), ultimately yielding two-dimensional Ti_3_C_2_ layers terminated with –F or –OH functional groups, which play a crucial role in determining MXene surface chemistry and application performance. In an effort to address the environmental concerns associated with the process, Carey and Barsoum developed an HF-etching method in which washing Ti_3_C_2_T_*x*_ with 0.05 M NaHCO_3_ reduced wastewater formation, enabled the recovery of fluorine as cryolite, and facilitated the preparation of a highly conductive delaminated MXene suspension.^63^

#### 2.1.2. *In situ* hydrofluoric acid etching

Although effective, the traditional HF etching pathway involves the use of highly concentrated HF and requires a series of processing steps, which are both very dangerous to the safety of the user and produce corrosive and toxic waste.^64^ To overcome these shortcomings, *in situ* HF etching plans are established as less harmful and greener options.^65^ At this moment, Cockreham *et al.* examined the formation condition of the byproduct AlF_3_·3H_2_O during the etching process using cobalt fluorides (CoF_2_/CoF_3_). Their research indicated that the establishment of this impurity is highly dependent on the environment of the reaction. Nevertheless, the SEM image of the CoF_3_/MAX sample (Fig. 2e) revealed that no AlF_3_·3H_2_O phase was visible, which proved that the optimal reaction conditions can be used to prevent the formation of undesired byproducts.^66^ On the same note, Ramalignam *et al.* reported the preparation of Ti_3_C_2_T_*x*_ MXene through an *in situ* HF route involving a LiF–HCl system. LiF in the present method was dissolved in 9 M HCl to produce HF according to reaction (6):

6 3HCl + 3LiF → 3LiCl + 3HF

Ti_3_AlC_2_ powder was then added after full dissolution, and the mixture was stirred at 45 °C for 24 h to selectively remove the Al layer in the MAX stage. The approach illustrates how a contained *in situ* HF generation is efficient in creating etching and minimizes the contact of concentrated HF. The overall etching mechanism is illustrated schematically in Fig. 2f.^67^ In another study, Liu *et al.* prepared MXenes from Ti_3_AlC_2_ under acidic fluoride conditions, followed by sequential centrifugation and repeated washing until a near-neutral pH was obtained. The final Ti_3_C_2_T_*x*_ product was collected after freeze-drying, producing layered MXene sheets with improved structural integrity.^68^

**Fig. 3:**
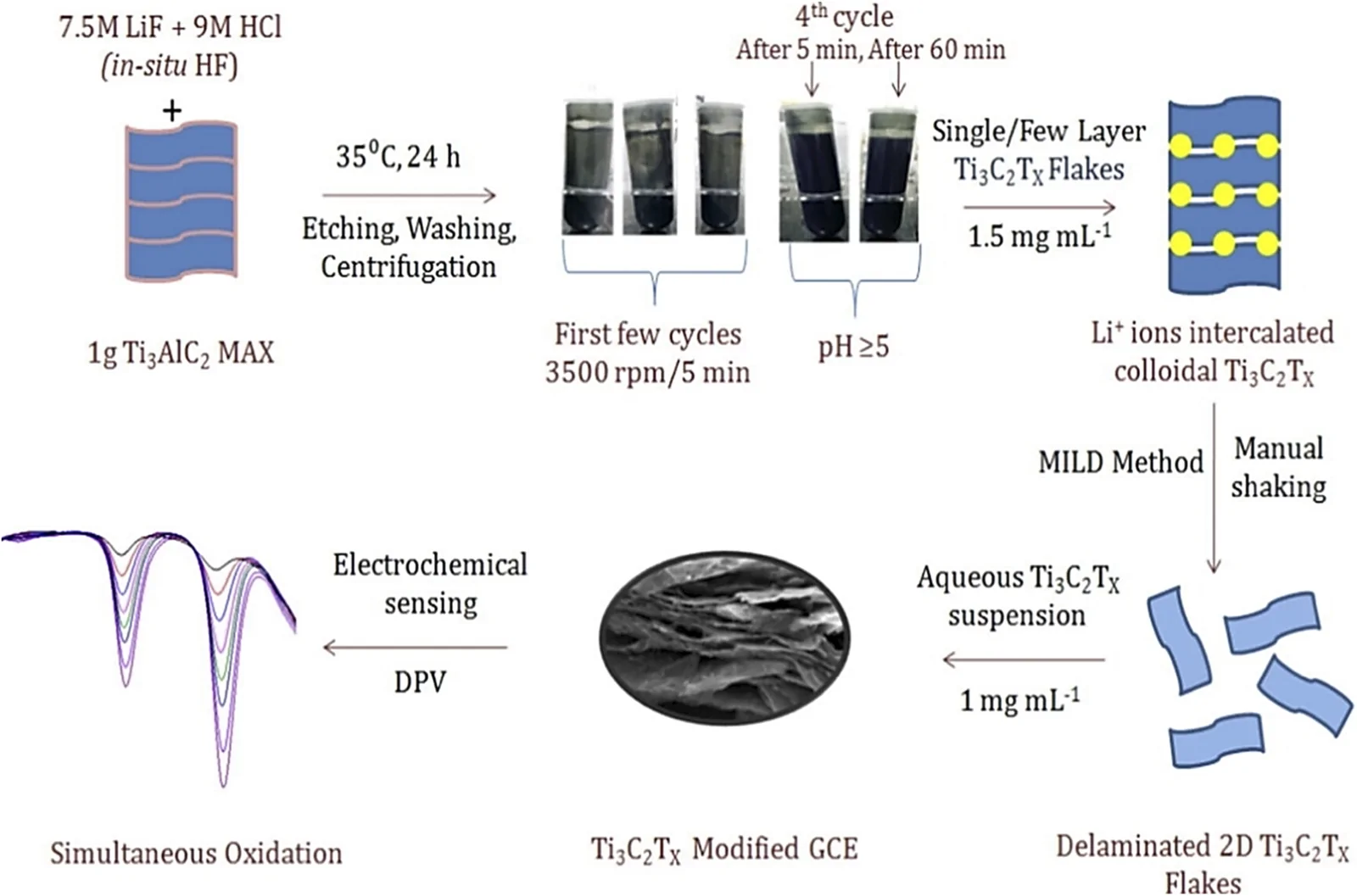
Preparation scheme of single/few layer Ti_3_C_2_T_*x*_ flakes using 7.5 M LiF/9 M HCl as the *in situ* HF etchant for carbamate detection. Reproduced with permission from ref. 69. Copyright 2021, Elsevier.

Furthermore, Sinha *et al.* demonstrated the successful synthesis of single- and few-layer Ti_3_C_2_T_*x*_ MXene using 9 M HCl and 7.5 M LiF molten fluoride salts.^69^ Remarkably, well-separated two-dimensional Ti_3_C_2_T_*x*_ nanosheets were obtained through simple manual shaking of the colloidal MXene suspension, highlighting the scalability and practicality of this *in situ* etching method.^69^ The preparation method of MXenes is demonstrated in Fig. 3. In another study, Alhabeb *et al.* reported a comprehensive overview of the synthesis protocols for Ti_3_C_2_T_*x*_ MXene using various etching methods, demonstrating that synthesis parameters significantly affect the flake size, yield, and material quality.^70^ Similarly, Liew *et al.* synthesized Ti_3_C_2_ MXene from the Ti_3_AlC_2_ MAX phase utilizing *in situ* hydrofluoric acid generated from NH_4_ and HCl. They comprehensively characterized the synthesized MXenes using FESEM, HRTEM, XRD, EDX, and BET, confirming successful etching and improved structural properties compared with different etching methods.^71^

#### 2.1.3. Electrochemical etching

**Fig. 4:**
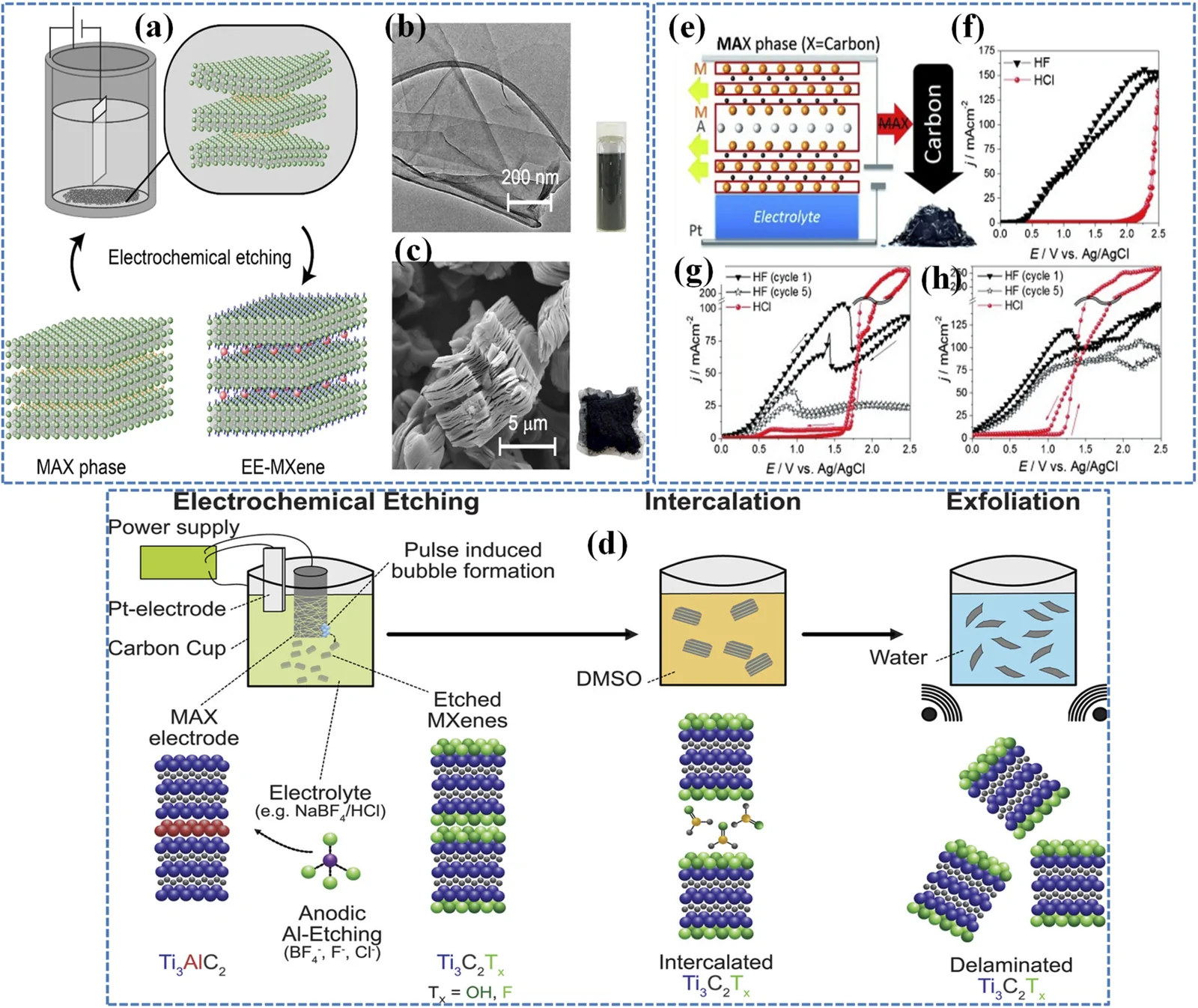
(a) Ti_3_C_2_ and Ti_3_CN MXenes were exfoliated from their corresponding MAX phases by electrochemical etching using HBF_4_ as the electrolyte. The performance of electrochemically etched MXene as electrodes. (b and c) SEM image. Reproduced from the Royal Society of Chemistry from ref. 74, which is open access and permits unrestricted use of materials under the terms of the Creative Commons CC-BY. (d) Electrochemical etching of Ti_3_AlC_2_ pellet electrodes in aqueous electrolytes: the set-up and workflow with schematic mechanisms to generate delaminated EC-MXene flakes. Reproduced from WILEY from ref. 75, which is open access and permits unrestricted use of materials under the terms of the Creative Commons CC-BY. (e) Graphic demonstration of the fabrication of the CDC from the MAX phase under room temperature conditions. Cyclic voltammograms acquired in HF (triangles) and HCl (circles) when (f) Ti_3_SiC_2_, (g) Ti_3_AlC_2_, and (h) Ti_2_AlC were employed as the anodes. Reproduced with permission. Reproduced with permission from ref. 76. Copyright 2026, WILEY.

An electrochemical etching method is proposed to obtain MXenes without the use of fluorine or alkali. In this approach, the weak M–A bonding interactions are exploited relative to the strong M–X bonding interactions, and the elements in the A-layer are selectively removed under the influence of an electric field.^72,73^ The etching process can be well controlled by controlling the etching time and potential applied, so A-sites can be selectively removed. In contrast to traditional HF-based methods, this electrochemical method does not introduce fluoride species at all, leaving MXene surfaces mostly with chloride, oxygen, and hydroxyl groups instead of fluoride.^72^ The pioneering study by Chan *et al.* provided an electrochemical method to synthesize Ti_3_C_2_ and Ti_3_CN MXenes using aqueous tetrafluoroboric acid as the electrolyte, which contains only a small amount of HF (Fig. 4a).^74^ The effect of applied potential and temperature on the etching kinetics was investigated and compared to chemical etching in HBF_4_. The following mechanism is suggested for the selective anodic dissolution of Al from Ti_3_AlC_2_ and Ti_3_AlCN involving the tetrafluoroborate ion. The formation of MXenes was confirmed by electron microscopy (Fig. 4b and c). It is worth noting, however, that the MXene flakes from electrochemical etching have larger lateral dimensions compared to chemically etched flakes, which is similar to the effects of suppressing the HF decomposition and the increased etching rate.

In a related advancement, the work of Ostermann *et al.* presented a scalable electrochemical protocol for MXene synthesis using a non-toxic and sustainable electrolyte composed of sodium tetrafluoroborate and hydrochloric acid (NaBF_4_/HCl) (Fig. 4d).^75^ The efficiency of etching is significantly improved using cathodic pulsing in pulse voltammetry, where hydrogen bubble formation restores electrochemical activity and is utilized to remove 2D sheets, thereby allowing for continuous etching with high *in situ* etch yields. In particular, electrochemical MXene (EC-MXene) yields of up to 60% were obtained without any byproducts. High purity and excellent quality of the EC-MXene were confirmed using chemical mapping using scanning electron microscopy (SEM/EDX) and surface termination analysis using X-ray photoelectron spectroscopy (XPS) and for the first time, low energy ion scattering (LEIS).

Earlier research work by Lukatskaya^76^ investigated electrochemical etching of MAX precursors, namely Ti_3_AlC_2_, Ti_3_AlC, and Ti_3_SiC_2_, using various electrolytes including 5 wt% NaCl, 10 wt% HCl, and 5 wt% HF, to produce carbon-derived carbon (Fig. 4e). The progress of etching was followed by cyclic voltammetry (Fig. 4f–h). At a certain voltage, it stimulates the breakup of M–A bonds, which begins the selective removal of the aluminum layers. As the voltage increases, however, the transition metal (M) layers are dissolved progressively until eventually amorphous carbon forms. The results of this investigation reveal that an accurate voltage window and reaction time control is critical for successful MXene synthesis to selectively remove A-layers while leaving behind M layers. The results highlight the importance of tuning electrochemical parameters such as electrolyte composition, applied potential, fabrication time and temperature to ensure controlled and efficient fabrication of MXenes.

#### 2.1.4. Hydrothermal processes

Hydrothermal methods have emerged as an effective and safer alternative for the synthesis of MXenes, primarily due to their ability to avoid the generation of extremely toxic vapors commonly associated with conventional HF-based etching routes.^77^ Under this method, salts containing fluoride, like ammonium fluoride (NH_4_F), are used to hydrothermally treat the Ti_3_AlC_2_ MAX phase, which causes the Al layers to be removed selectively and Ti_3_C_2_T_*x*_ MXene to be formed. This approach has been applied to other MXenes besides Ti_3_C_2_T_*x*_, such as Ti_3_C_2_ and Nb_2_C, which have been successfully synthesized by hydrothermal etching using NaBF_4_ with HCl, thus removing all traces of HF entirely.^77,78^

Hydrothermally prepared MXenes have several desirable structural properties compared to those made by traditional methods such as HF etching. These are improved surface termination removal, increased interlayer spacing, improved delamination behavior, and higher *c*-lattice parameters.^79^ All these attributes emphasize the efficiency of hydrothermal processing in the modification of the structural and physicochemical characteristics of MXenes towards advanced uses.

**Fig. 5:**
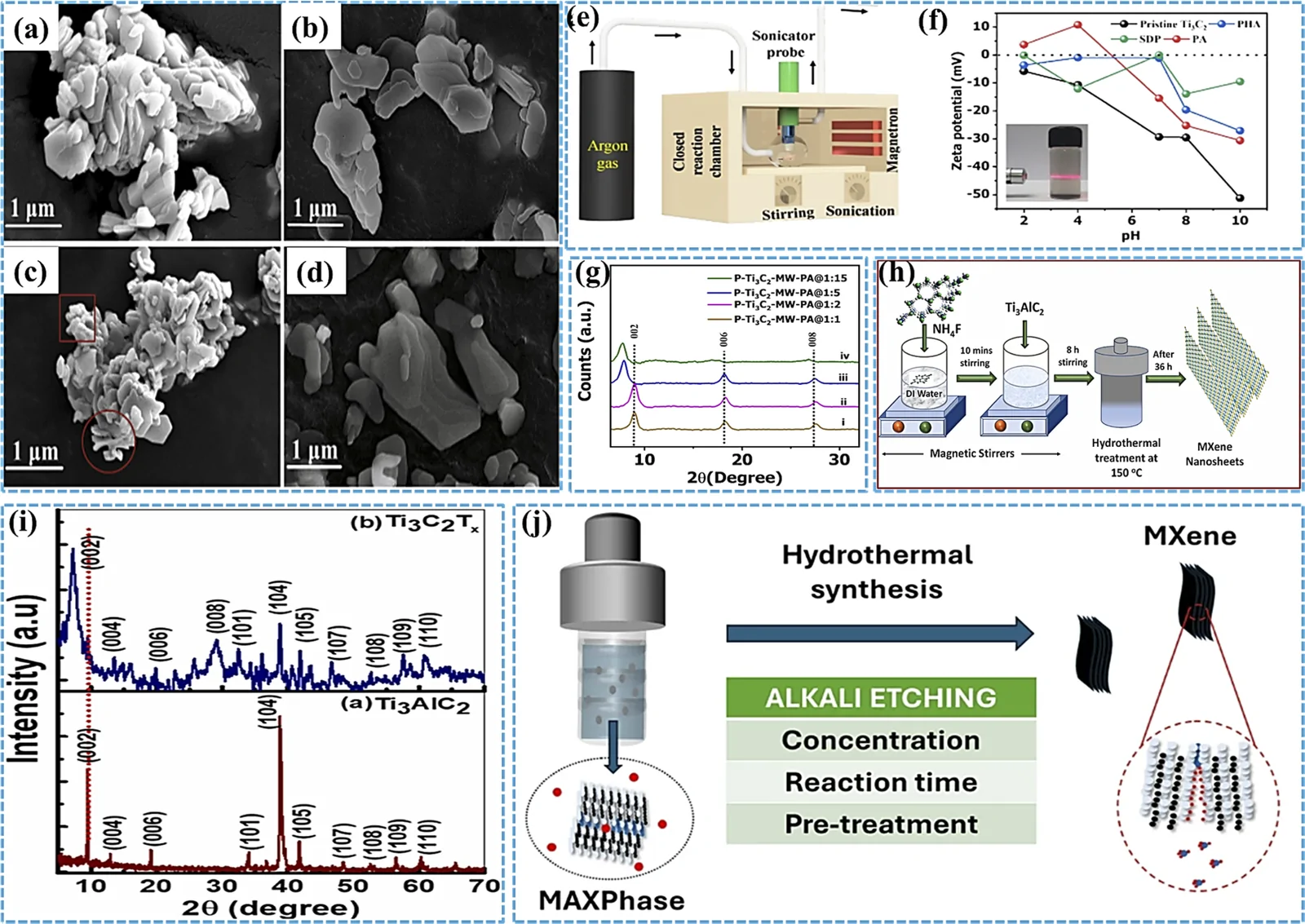
(a–d) SEM images of Mo_2_CT_*x*_–N MXene after heat treatment at 200 °C in Ar (a), 500 °C in Ar (b), 700 °C in Ar (c) and at 200 °C in air (d). Reproduced from Elsevier from ref. 80, which is open access and permits unrestricted use of materials under the terms of the Creative Commons CC-BY. (e) Simple sketch of the MW heating chamber, (f) zeta potential plot of pristine Ti_3_C_2_T_*x*_ and different doping sources at different pH values and (g) XRD patterns of P–Ti_3_C_2_–MW-PA at different ratios: (i) P–Ti_3_C_2_–MW-PA@1 : 1, (ii) P–Ti_3_C_2_–MW-PA@1 : 2, (iii) P–Ti_3_C_2_–MWPA@1 : 5 and (iv) P–Ti_3_C_2_–MW-PA@1 : 15. Reproduced with permission from ref. 81. Copyright 2020, Royal Society of Chemistry. (h) Schematic for the synthesis of MXene (Ti_3_C_2_T_*x*_) from its MAX phase using the hydrothermal method and (i) XRD diffractograms of MAX phase and MX nanosheets. Reproduced from Elsevier from ref. 82, which open access and permits unrestricted used of material under the terms of the Creative Commons CC-BY. (j) Graphical representation of MXene synthesis *via* hydrothermal synthesis. Reproduced from Springer Nature from ref. 83, which is open access and permits unrestricted use of material under the terms of Creative Commons CC-BY.

Even though the use of fluoride salt does not cause any significant changes in the basic layered structure of MXenes, it is an important factor that determines the most favorable synthesis conditions. In this context, Guo *et al.* systematically investigated the use of LiF, NaF, KF, and NH_4_F in the presence of HCl for the hydrothermal synthesis of Mo_2_CT_*x*_ MXene. All synthesized samples exhibited similar layered morphologies; however, minor variations in Mo content (ranging from 3.6 to 7%) were observed. Notably, the optimal synthesis temperature was found to be strongly dependent on the solubility of the fluoride salt and the hydrated ionic radius of the corresponding cations, as evidenced by SEM analysis (Fig. 5a–d).^80^ A similar principle applies to other non-conventional synthesis routes, such as microwave-assisted methods, where microwave irradiation is used instead of ultrasonic treatment following the etching process (Fig. 5e and f). These approaches further demonstrate that the weakening of the M–X bond in MAX phases can be achieved through different energy-assisted pathways. Consequently, the influence of such methods on the resulting MXene structure and surface chemistry must be carefully considered; Fig. 5g shows the crystallinity of the prepared membrane.^81^

Recently, Tyagi *et al.* reported the successful synthesis of Ti_3_C_2_T_*x*_ MXene *via* a controlled hydrothermal route using NH_4_F as a mild etchant.^82^ In their method, ammonium fluoride was dissolved in deionized water, followed by the gradual addition of Ti_3_AlC_2_ under continuous stirring to form a dark suspension. The mixture was subsequently subjected to hydrothermal treatment at 150 °C for 36 h in a Teflon-lined autoclave. After completion of the reaction, the product was thoroughly washed with NaOH solution and deionized water, vacuum-filtered, dried at low temperature, and ground to obtain fine MXene powder. The overall synthesis procedure is illustrated in Fig. 5h, and the crystallinity of the prepared MXene was evaluated by XRD (Fig. 5i). In addition to fluoride-based systems, alkali-assisted hydrothermal etching has also been explored as a greener alternative. Kulkarni *et al.* reported a unique one-pot hydrothermal method for the fabrication of multilayer Ti_3_C_2_OH MXene by etching Ti_3_AlC_2_ using relatively non-toxic potassium hydroxide solutions. In this study, the quality and structural integrity of the resulting MXene were systematically evaluated as a function of alkali concentration, precursor pre-treatment, and total reaction time, highlighting the versatility and tuneability of hydrothermal routes for MXene synthesis (Fig. 5j).^83^

#### 2.1.5. Urea glass route

One such general bottom-up synthesis approach to MXene-like materials is the urea-glass method, where metal halide sources (*e.g.*, MoCl_5_) react with urea in alcohol media, executing a homogeneous glassy forerunner. This precursor, in turn, is subjected to a carefully monitored pyrolysis process in the presence of an inert atmosphere to give rise to nanocrystalline metal carbides or nitrides. Notably, the molar ratio between urea and the metal precursor is also important in deciding which phase is formed predominantly, either carbide or nitride, making this method a highly reliable way to make MXene-like materials when hazardous etchants are avoided.^84,85^ Under this method, nanocrystalline single-phase Mo_2_N and Mo_2_C MXenes have been prepared as well using the urea-glass composition method, where the urea-to-metal ratio has been varied systematically. At the molecular scale, it is synthesized by means of dissolving MoCl_5_ in ethanol and then reacting it with urea to produce molybdenum orthoester intermediates. These intermediates are then subjected to gelation, and the gel is subjected to heating at a temperature of around 800 °C in a non-reactive atmosphere, which finally forms a typical silver-black nanocrystalline precipitate.^85,86^ Urea is a compound that serves as a source of carbon and nitrogen and as a stabilizer. This greener reaction results in large surface areas of MXenes, which are highly effective as catalysts. It is worth noting that urea has a multifunctional role in such a process, as it can be used as a source of carbon and nitrogen, and as a structural stabilizer, which helps to obtain a homogeneous phase during pyrolysis. The result of this more environmentally friendly and more eco-friendly synthesis pathway is MXene-like materials with a high specific surface area, which translates to high catalytic performance and efficiency. Thus, urea-based bottom-up approaches can act as a viable alternative to traditional etching pathways towards the scalable and sustainable synthesis of MXenes and other two-dimensional compounds.^87,88^

### 2.2. Structure–property relationships of MXene nanosheets

The basic structure of MXenes is similar to that of parent MAX phases: transition metal carbide/nitride layers alternate with A-group elements. The A element is selectively etched to release M_*n*+1_X_*n*_ nanosheets capped with functional groups, which have a high aspect ratio, large surface area, and specific electronic properties. The electronic properties are highly affected by the d-orbitals of the transition metal, which is present in the MXenes; for many of them, partially filled d-band states result in a metallic electronic behavior, with variations depending on both the metal composition and the surface functionalization, as revealed by DFT calculations. For example, the metal-like Ti_3_C_2_ becomes semiconducting upon functionalization with –OH and –O groups due to the opening of the band gap. The functional extension of the MXenes comes with the introduction of other elements in the structure (substitutional doping/alloying) as well as with ternary/quaternary MXenes (such as Mo_2_TiC_3_ and TiVNbC), which are characterized by their modified band structures, magnetic properties, and charge-transport function, useful for advanced electronic and spintronic systems.

#### 2.2.1. Surface chemistry

Surface chemistry is a characteristic feature of MXenes. Basal planes and edges will be terminated by –OH, –O, –F, and –Cl groups depending on the route of the synthesis during etching and post-treatment.^89^ These terminations profoundly impact hydrophilicity, electrochemical activity, and interactions with guest species. –OH and –O groups enhance hydrophilicity and hydrogen bonding, making MXenes excellent for aqueous dispersions and composite interfaces. Surface terminations pseudocapacitively contribute to charge storage in electrochemical applications by virtue of redox-active sites. The oxygen-terminated surface of MXenes shows increased capacity and cycling stability in battery systems because it has greater electrostatic interactions with Li^+^, Na^+^, or K^+^ ions,^90^ while the fluorinated surface can block ion transport by its weaker binding and steric influence. By controlling surface terminations, this gives a route to fine-tune MXenes for specific applications.

#### 2.2.2. Thickness

This is an important factor in MXene properties. If the exfoliation process reduces the material to a monolayer or few-layer thickness, it changes its optical, electrical, and mechanical properties from those of the bulk material. The electrical conductivity of the thin flakes is enhanced by the reduced interlayer resistance and effective charge delocalization, which are appropriate for transparent conductive film, antenna, and supercapacitor electrodes.^91^ They are thin enough to be flexible like wearable electronics and soft sensors. Thinner sheets have a smaller diffusion path, resulting in better rate capability and greater accessible surface area of ions in electrochemical devices. But when the ratio is too high, restacking and aggregation will occur, which should be prevented by using interlayer spacers or surfactants.

Most of the MXene nanosheets are chemically processed, which inevitably introduce defects such as vacancies, grain boundaries, and edge dislocations. Defects can be charging traps or structural weakness but can also be active sites for catalysis or sensing. Vacancies on M or X sites not only increase electrocatalytic activity but also introduce under-coordinated atoms, which can support reaction kinetics. The sensitivity in chemical sensing is enhanced by the edge defects, which increase the surface reactivity against analytes. But high defect density has a negative effect on conductivity, mechanical strength, and long-term stability. To control defect levels, a post-synthesis process involving annealing, chemical healing or controlled doping is used.

#### 2.2.3. Crystallinity

This affects the properties: highly crystalline flakes will have uniform electronic transport and fewer scattering centers, which is useful for field-effect transistors or electromagnetic shielding.^92^ On the other hand, amorphous or partially disordered MXenes provide more catalytic/adsorption-ready active sites. For multilayered stacks, the interactions between the layers, which involve van der Waals forces and electrostatic attraction, have an effect on the performance, but restacking causes a decrease in available surface area, inactivity of active sites, and diffusion restrictions for ions. To control interlayer spacing, intercalation of organic molecules, ions, or polymers; hydrogel and composite formation; and pillaring with carbon nanotubes, graphene oxide, and silica nanoparticles are the strategies used that maximize ionic accessibility but maintain the structural integrity.

#### 2.2.4. Anisotropy

This is an important characteristic of MXene nanosheets. The conductivity is generally one to several orders of magnitude higher in-plane than out-of-plane, as a result of the extended conjugated network of metal–carbide/nitride bonds, which makes MXenes suitable for planar supercapacitors or interconnects.^93^ Mechanical anisotropy is also present, showing relatively strong in-plane Young's modulus and tensile strength, but relatively weak cohesion between the layers, which is good for exfoliation but is not good for composite stability.

#### 2.2.4. Roughness, hydrophilicity, thickness, and pore size

These are governed by surface chemistry. The surface chemistry of MXenes can be adjusted through the use of the functional groups (T_*x*_) deposited during the synthesis.^89^ These terminations play a key role in hydrophilicity, colloidal stability, electrochemical activity, and interfacial interactions. Hydrophilicity is particularly important, allowing MXenes to be dispersed in water without the need for a surfactant, whereas hydrophobic graphene or TMDCs require a surfactant for dispersion. Oxygen- and hydroxyl-terminated surfaces are more reactive electrochemically and are able to facilitate charge transfer; hydroxyl groups can improve biocompatibility and promote biomolecule adsorption for biosensing or drug delivery. The relative amounts of –F, –OH, and –O groups can be varied by changing the etching environment, the etching time, the etching temperature, or the washing protocol, thus controlling surface chemistry and the resulting properties.^94^ Thermal annealing and chemical treatments selectively remove fluorine terminations and increase coverage with oxygen or hydroxyls, which is beneficial for conductivity and chemical stability. Tunable hydrophilicity influences polymer interactions, which influences mechanical strength, conductivity, and processability in hybrid materials. However, MXenes are inherently hydrophilic and can be chemically modified by alkyl silanes and/or polymer grafting to enable the introduction of hydrophobic properties to separate oil and water and/or apply hydrophobic coatings.^95^

#### 2.2.5. Surface termination composition

This is dominated by –O, –OH, and –F groups that are being added during the etching process. The terminations define physical, chemical, and electronic properties, which in turn influence the performance of the material in energy storage, catalysis, sensing, and environmental remediation. Types, distribution, and density of surface functional groups can be modulated by varying synthesis parameters such as etching duration, temperature, and post-etching treatments.^96^ Advanced spectroscopic methods (XPS and NMR) have been used to understand surface termination chemistry, which is found to be very sensitive to synthesis conditions. The –O, –OH, and –F groups in the synthesized Ti_3_C_2_T_*x*_ after HF etching usually vary in relative abundance depending on the concentration, duration, temperature, and washing and annealing procedures of the etching process.^60^ Hydroxyl and oxygen terminations are preferred under conditions of lesser severity and are associated with improved hydrophilicity and electrochemical activity, while overfluorination has a negative impact on conductivity and chemical stability. Post-synthesis modifications are also possible, such as thermal annealing, chemical etching or plasma treatment that can selectively remove or convert surface groups, increasing conductivity and the environmental stability;^97^ (annealing in an inert or reducing atmosphere removes fluorine and increases oxygen-based terminations). Surfaces can be functionalized *via* chemical treatments with amine or thiol for sensing or catalytic behavior.

The hydrophilicity is very high because of the presence of polar surface terminations like –OH and –O. The typical contact angle of these materials is less than 20°, meaning they are superhydrophilic.^55^ This will make it easier to disperse in an aqueous solution where no surfactant is needed to form a stable colloidal suspension. The hydrophilic aspect increases the interaction between solvents and electrolytes, which is suitable for use in the aqueous based supercapacitors and batteries. The surface energy has been estimated to be 70–80 mJ m^−2^, which is higher than that of graphene (46 mJ m^−2^) but lower than that of graphene oxide (62 mJ m^−2^).^98^ The surface chemistry and layer structure are very hydrophilic, which allows for efficient ion intercalation and outstanding pseudocapacitive energy storage. The occurrence of many surface terminations gives many active sites for faradaic redox reactions, and the interlayer spacing opens up many channels for hydrated ions. This allows for fast and reversible charge transfer that is the basis of pseudocapacitive behavior. The synergic combination of electrical double-layer capacitance and pseudocapacitance from fast redox reactions at surface terminations offers high energy and high-power density charge storage. Layered morphology provides easy diffusion of ions in interlayer galleries, decreases diffusion resistance, and improves rate capabilities. The interlayer spacing is tunable and can be optimized according to the size of the ion and the solvent environment.

#### 2.2.6. Reactive surface terminations

Reactive surface terminations offer versatile platforms for chemical functionalization and surface modification, enabling precise tailoring of physicochemical properties.^99^. –O, –OH, and –F groups provide abundant sites for covalent and non-covalent interactions, facilitating functionalization strategies including covalent attachment of organic molecules, polymer grafting, and decoration with metal or metal oxide nanoparticles. These changes improve the stability, selectivity, and functionality of MXenes for chemical sensing, catalysis, and energy storage applications. Covalent grafting of alkyl chains greatly promotes dispersibility and stability in organic solvents while strengthening compatibility with hydrophobic polymer matrices, which allows for the fabrication of MXene-based composites with excellent mechanical strength and electrical conductivity.^100^ Active surface area is increased, and charge-transfer kinetics are improved by decoration with noble metal nanoparticles (Au, Pt, and Pd), which will give synergistic effects and make the material more catalytic and selective.^101^ This strategy takes advantage of the conductive and chemically active platform of MXenes to stabilize and disperse the metal nanoparticles, thus avoiding aggregation and enhancing catalytic efficiency.

### 2.3. Fabrication of MXene membranes

The unique attributes of 2D nanomaterials such as MXenes, namely their tunable structure, versatile surface properties, and controllable interlayer spacing have positioned them as highly attractive building blocks for advance membrane systems.^102^ MXene membranes can be designed based on a set of pure nanosheets or laminated nanostructures, both of which play a crucial role in determining the performance of membranes. However, there are several issues to overcome, which are the generation of MXene materials at a large scale, the optimization of the method of membrane fabrication, the stability of the structure under the work conditions, and the balance between permeance and selectivity. Therefore, the logical design of MXene lamellar membranes as working molecular sieves necessitates accurate control over film density, thickness, mechanical strength, and interlayer spacing to obtain defect-free, ultrathin, and mechanically stable membranes with regulated pore size.

**Fig. 6:**
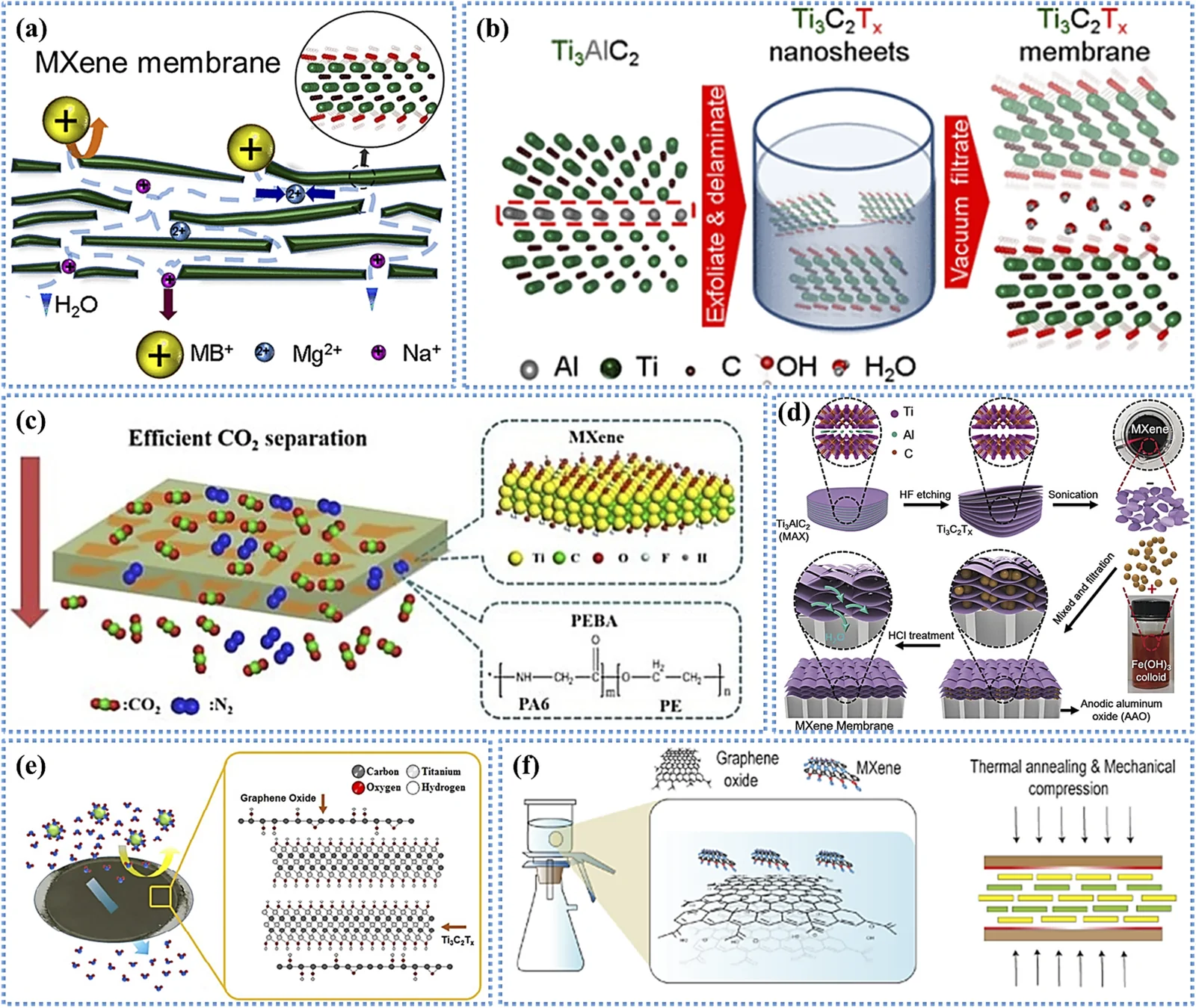
(a) Schematics showing differential permeation of cations through a Ti_3_C_2_T_*x*_ membrane, and total rejection of large MB^+^: (b) preparation, structure, and images of Ti_3_C_2_T_*x*_ membranes. Reproduced from the American Chemical Society from ref. 37, which is open access and permits unrestricted use of materials under the terms of the Creative Commons CC-BY. (c) Schematic diagram of the Pebax-based mix matrix membrane with MXene nanosheets. Reproduced with permission from ref. 106. Copyright 2019, Asian Chemical Editorial Society. (d) MXene membrane preparation. Reproduced with permission from ref. 107. Copyright 2017, Wiley. (e) Photograph of the Ti_3_C_2_T_*x*_–GO composite membrane for water filtration (left) and the membrane structure (right) and the corresponding schematic illustration. Reproduced with permission from ref. 108. Copyright 2017, American Chemical Society. (f) Schematic of the dynamically cross-linked GO/MXene membrane. Reproduced from American Chemical Society from ref. 109, which is open access and permits unrestricted use of materials under the terms of the Creative Commons CC-BY.

The first successful application of MXene materials in the membrane separation method was reported by Ren *et al.*, marking the beginning of MXene-based membrane technologies (Fig. 6a).^37^ They prepared free-standing MXene membranes through the vacuum-filtering of delaminated Ti_3_C_2_T_*x*_ nanosheets (Fig. 6b) dispersed in dilute deionized water on a commercial polyvinylidene fluoride (PVDF) support that had a pore size of 450 nm. After completing drying, the resulting MXene films with tunable thicknesses ranging from several hundred nanometers to a few micrometers were easily peeled off from the PVDF substrate and found to be highly flexible, mechanically strong, and structurally intact. In addition to freestanding forms, the integration of MXene nanosheets as fillings or additives to traditional polymer frameworks has become one of the feasible options to achieve the desired level of scalability and to minimize the price of fabrication at the same time. Critical parameters like filler dispersion, interfacial compatibility, and long-term stability should be effectively optimized in the mixed-matrix membranes (MMMs) to maximize the benefits of MXene materials.^103,104^ Much more sophisticated methods of fabrication have been used to prepare MMMs, such as blade casting and spin coating. Indicatively, solvent-assisted deposition methods have been used whereby MXene nanosheets with lateral dimensions of 500–1000 nm and thicknesses of around 1–2 nm were spin-coated onto chitosan (CS) surfaces to produce uniform layers of membranes.^105^ Similarly, Liu *et al.* demonstrated fabrication of MXene mixed-matrix membranes through a polymeric matrix approach to improve the performance of gas separation, and processability of the membrane (Fig. 6c).^106^ SEM was used to characterize the fabricated MXene membrane morphology. Besides, Ding *et al.* described a new group of laminar MXene membranes that consisted of Ti_3_C_2_T_*x*_ nanosheets, as shown in Fig. 6d that were formed by vacuum filtration of etched Ti_3_AlC_2_ precursors onto porous supports. Due to the presence of a large number of surface functional groups (–O, –OH, and –F), these membranes demonstrated increased hydrophilicity and adjustable interlayer spacing, and thus became a good candidate to be used in selective separation processes.^102,107^ Besides that, Kang *et al.* synthesized an ultrathin Ti_3_C_2_T_*x*_ graphene oxide (GO) composite membrane by a simple mixing of both GO and MXene suspensions and vacuum filtration onto porous materials, making the membrane about 90 nm in thickness (Fig. 6e). This hybrid strategy effectively combined the mechanical stability of GO with the high permeability of MXenes.^108^ Similarly, GO–MXene composite membranes were prepared by dispersing GO (2 g L^−1^) and Ti_3_C_2_T_*x*_ MXene in deionized water, followed by vigorous mixing to ensure homogeneity (Fig. 6f). The resulting suspensions were vacuum filtered onto polyethersulfone (PES) supports with a pore size of 100 nm. Notably, self-cross-linked GO–MXene membranes were obtained through ambient drying, followed by thermal treatment at 120 °C under vacuum for 24 h, which significantly improved membrane stability and interlayer cohesion.^109^Table 1 summarizes the fabrication/modification, synthesis, applications, and key advantages of the various reported MXene and MXene-based membranes.

**Table 1:** Summary of various MXene-based membranes and their key performance, synthesis, and modifications

Membrane	Fabrication/modification	Method	Application (removal)	Key properties	References
Ti_3_C_2_T_*x*_	PVDF	Vacuum assisted filtration	Li^+^, Na^+^, K^+^, Mg^2+^, Ca^2+^, Ni^2+^, and Al^3+^	Tunable thickness, high flexibility, strong mechanical stability, hydrophilic surface, electrically conductive, ultrafast water flux, and charge and size-selective ion rejection	37
MXene/Pebax	Poly (ether-amide) block copolymer	Vacuum filtration	CO_2_	High permeance and selectivity, and is useful for efficient CO_2_ capture	106
Ti_3_C_2_T_*x*_ with the functional groups T (O, OH, and/or F)	Anodic aluminum oxide (AAO)	Vacuum filtration	—	Extremely high water permeance along with good stability	107
Ti_3_C_2_T_*x*_–GO	Graphene oxide	Vacuum filtration	Methyl red, methylene blue, Rose Bengal and brilliant blue	Controlling size and charge separation; high rejection	108
GO–MXene composite	Graphene oxide	Vacuum filtration	Allura red, Victoria blue, and methyl blue	Resists swelling and maintains strong dye and salt rejection over long periods	109
TiO_2_–MXene mesoporous	TiO_2_ hydrosol	Dip-coating/sol-coating	—	Maintains strong and stable rejection of dyes and salts over time	114
Ti_3_C_2_T_*x*_-PES	Polyethersulfone	Filtration at 0.2 MPa	Congo red inorganic salts	Excellent hydrophilicity, high water flux, and effective dye rejection	115
g-C_3_N_4_	2D g-C_3_N_4_	Layer-by-layer	Rhodamine B and Evans blue	Self-supporting spacers and artificial nanopores, enabling ultralow-friction water transport with high permeance and rejection	116
			Cytochrome C Au NPs		
Ti_3_C_2_T_*x*_	Ag-NP	Self-reduction	BSA *E. coli* growth bacteria	Self-supporting spacers and artificial nanopores, enabling ultralow-friction water transport with high permeance and rejection of 3 nm molecules	117
TiO_2_@MXene	PES-TiO_2_	Hydrothermal oxidation	BSA	High flux and 95% BSA rejection, with excellent UV-driven self-cleaning and antifouling performance	118
			Sodium alginate and humic acid yeast		
MXene	PAN substrate and PEI	Vacuum filtration	MgCl_2_	Achieving high water permeance and excellent salt rejection, making it effective for nanofiltration and forward osmosis desalination	119
MXene	CA	Phase inversion	—	High water flux (12.2 L m^−2^ h^−1^) and excellent fouling resistance; it is effective for seawater treatment and wastewater applications	120
MGOm	GO	Vacuum filtration	Al^3+^	Conductive, voltage-responsive nanochannels, allowing tunable ion transport	121
MXene/BN@PDA/PEI	BN@PDA/PEI	Vacuum filtration	Oil–water	Maintains stable interlayer spacing (14.7 Å) and shows strong anti-swelling, hydrophilicity, and antifouling	122
MXene–carbon nanotube ultrathin	CNT	Self-designed pressure-assisted filtration	Congo red, methyl orange, rhodamine B, MgSO_4_, Na_2_SO_4_, MgCl_2_, and NaCl	Showing high water permeance, excellent rejection, and strong anti-swelling, making it effective and durable for water purification	123
CTS–MXene/PAN composite	Chitosan	Vacuum filtration	Congo red	Providing fixed spacing (14.33 Å), ultra-high flux, dye rejection, excellent protein filtration, and long-term reusability	124
MXene-porphyrin-PVA hydrogel	Polyvinyl alcohol (PVA) and porphyrin	Vacuum filtration	RhB	High solar-driven water evaporation rate and photodegradation efficiency	125
MXene@TiO_2_/g-C_3_N_4_	Graphitic carbon nitride (g-C_3_N_4_) and TiO_2_ NPs	Vacuum-assisted self-assembly	C/W, T/W, D/W, DM/W, and H/W	Superior self-cleaning ability, excellent separation and catalytic degradation capacity	126
KH570–TiO_2_/MXene/PAN	PAN and KH-570 modified TiO_2_	Electro spraying	Methylene blue	Excellent photocatalytic degradation performance to organic dyes and a high evaporation rate to water	127

**Fig. 7:**
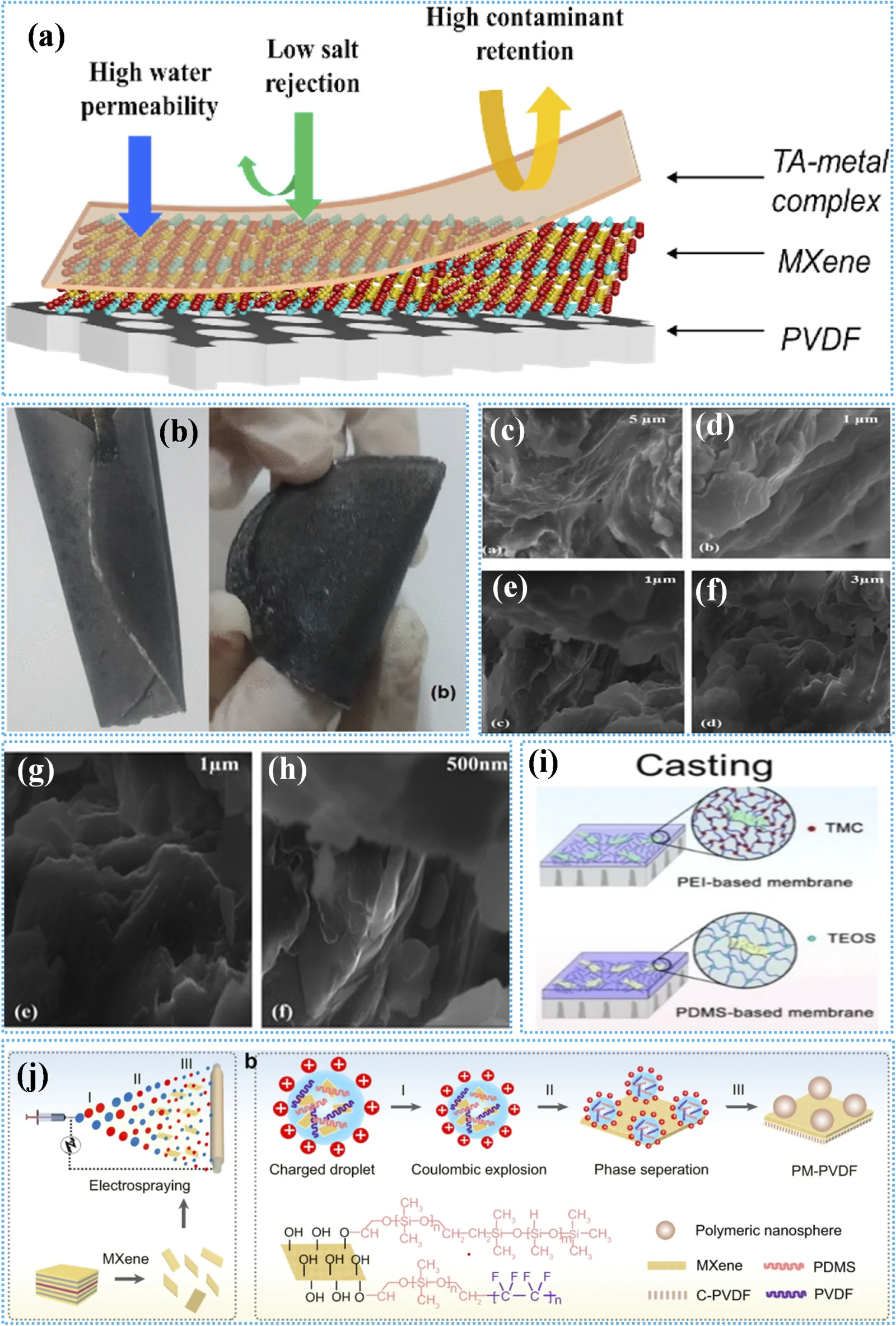
(a) Graphical illustration of a tannic acid–metal complex modified MXene membrane for contaminant removal. Reproduced with permission from ref. 110. Copyright 2021, Elsevier. (b) Silane-grafted MXene-incorporated PVA/GA membranes. SEM images of silane-grafted MXenes: (c and d) MNOCS, (e and f) MODCS, and (g and h) MTPCS. Reproduced from American Chemical Society from ref. 111, which is open access and permits unrestricted use of materials under the terms of the Creative Commons CC-BY. (i) Electrospraying of MXene nanosheets on polymer supports. Reproduced with permission. Reproduced from Springer Nature from ref. 112, which is open access and permits unrestricted use of materials under the terms of the Creative Commons CC-BY. (j) Casting MMMs with functionalized MXene nanosheets on PAN substrates. Reproduced with permission. Reproduced with permission from ref. 113. Copyright 2017, Elsevier.

Tong *et al.*^110^ synthesized MXene membranes by the vacuum-assisted filtration technique. A diluted MXene suspension (1 mg mL^−1^) was filtered through a PVDF support membrane (47 mm diameter), and the thickness of the obtained MXene layer was controlled *via* the volume of the filtered suspension. The filtered composite membrane was then air-dried. Subsequently, a two-step surface modification was carried out to functionalize the MXene layer. The reaction solution was applied to the side of the membrane coated with the MXenes. First, 30 mL of a metal salt solution (3 mM FeCl_3_, CuCl_2_, or ZnCl_2_, pH ∼7) was added under moderate shaking for 0.5 minutes. Then, without decanting, 30 mL of a tannic acid (TA) solution (3 mM) was introduced and shaken for an additional 1 minute. Finally, the mixed solution was then decanted, and the modified membrane was rinsed with DI water before characterization. The schematic representation of the prepared membrane is presented in Fig. 7a. In a similar approach, Yousef *et al.* synthesized MXene–polymer membranes with PVA as a binder (Fig. 7b).^111^ To prepare a 1% PVA solution, 1 g of PVA was dissolved in 100 mL of water at 90 °C for 3 h. Thereafter, 0.1 g pristine MXene, MODCS, MNOCS, and MTPCS were respectively added to 100 mL of 1% PVA solution and stirred for 2 h, followed by filtration using an MCE membrane filter (0.22 µm) to obtain homogeneous dispersions and deposit them as a uniform layer (Fig. 7c–h). In addition, free-standing cross-linked membranes were separately fabricated by adding 0.5 mL of glutaraldehyde (GA) into 4 mL of 1% PVA, dispersion of 0.1 g of each MXene material, casting into Teflon Petri dishes (diameter 70 mm), and finally curing at 40 °C for 3 h. In another recent study, Zhang *et al.*^112^ successfully prepared MXene–polymer MMMs using the simple electrospray method (Fig. 7i) and employed them in desalination *via* membrane distillation. Notably, the functionalization of the surfaces on the MXene nanosheets changed the surface wettability from hydrophilic to superhydrophobic, thereby giving rise to the photothermal effect as well as high water-repellency to the membranes. Similarly, to improve the compatibility of MXenes with the polymer solutions (PEI or polydimethylsiloxane), Hao *et al.* first functionalized MXene nanosheets with four different functional groups.^113^ The resulting composite suspension was then drop cast on a polyacrylonitrile (PAN) support layer (Fig. 7j), thereby fabricating MMMs for use in solvent nanofiltration applications.

### 2.4. Structure–transport relationship in MXene membranes

**Fig. 8:**
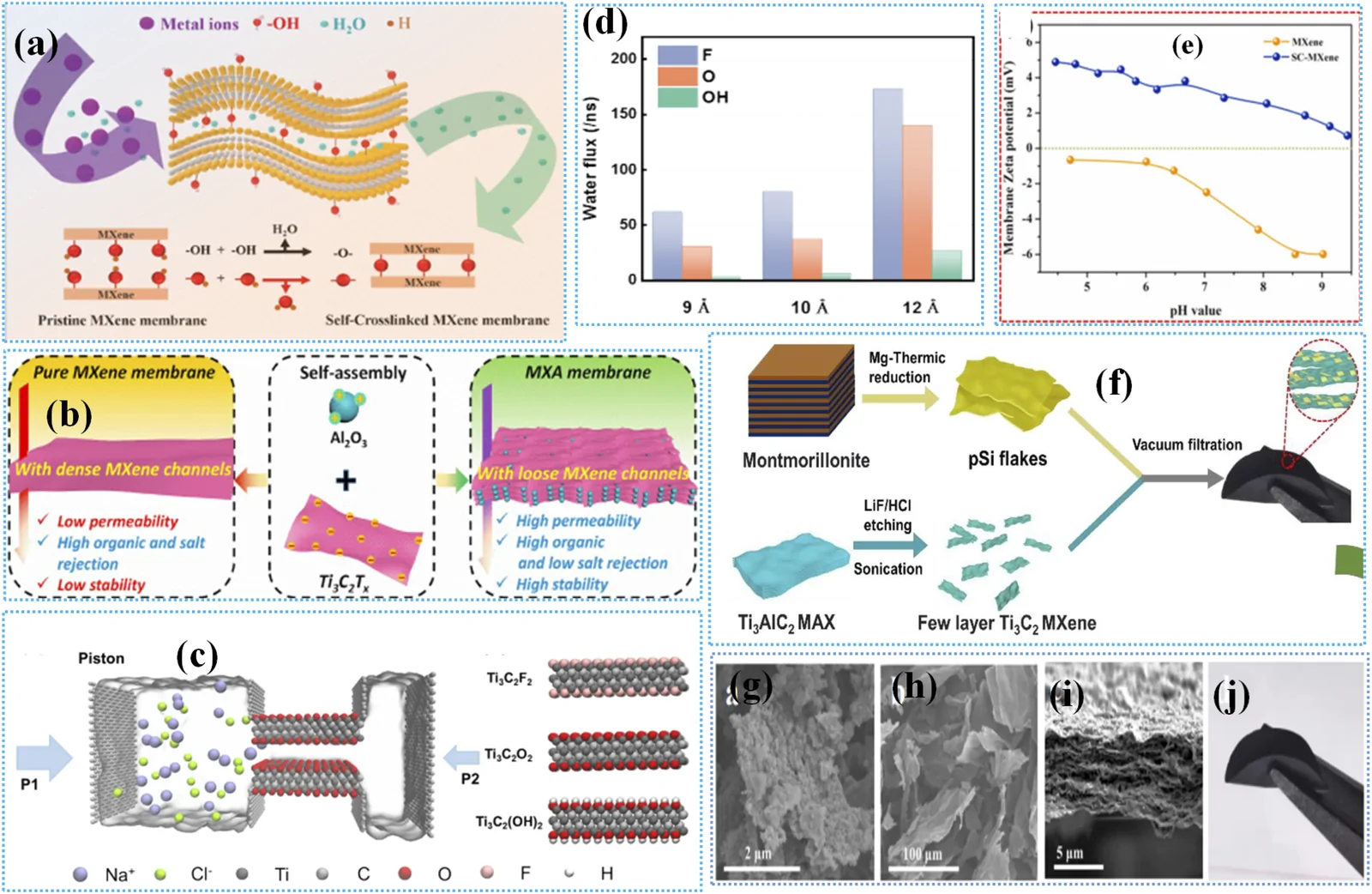
(a) Self-crosslinking of MXene membranes *via* condensation of –OH groups on adjacent nanosheets. Reproduced with permission from ref. 139. Copyright 2019, American Chemical Society. (b) Schematic illustration of interlayer spacing by adjusting the ratio of MXenes to Al_2_O_3_ in the membrane assembly. Reproduced with permission from ref. 140. Copyright 2021, Elsevier. (c) Simulation model for the desalination process and MXene nanosheets with different termination groups. (d) Water flux through a nanochannel with respect to different interlayer distances. Reproduced with permission from ref. 144. Copyright 2022, Elsevier. (e) Surface charge characteristics expressed as zeta potential for pristine and self-cross-linked MXene (SC-MXene) membranes. Reproduced with permission from ref. 119. Copyright 2021, Elsevier. (f) Schematic illustration for the preparation of pSi/MXene-2 : 1 films. (g, h, and i) SEM images of pSi, MXene, and pSi/MXene-2 : 1 film and (j) a photograph of the pSi/MXene-2 : 1 film. Reproduced with permission from ref. 161. Copyright 2023, Elsevier.

MXene materials are two-dimensional transition metal carbides and nitrides with a tunable surface chemistry and layered structure.^128^ In this structure, the nanosheets are hydrophilic and exhibit negatively charged surfaces, mechanical strength, and strong metallic bonds between them. Moreover, the gaps in the stack are controllable and form molecular sieves that prevent hydrated ions from entering the stack while facilitating fast water transport, making these materials very promising for next-generation purification. A precise control of the interlayer spacing of MXene-based membranes is one of the critical characteristics which directly influences the purification performance.^129^ The natural nanolaminated MXenes offer two-dimensional nanochannels that can be tailored by varying the surface chemistry and intercalation of ions or molecules, as well as environmental conditions and mechanical pressure.^130,131^ In comparison with many other 2D materials, this tunability is a unique property of MXene membranes. In purification processes like nanofiltration and reverse osmosis, the membrane can separate water molecules (hydrated radius ∼2.75 Å) from the larger hydrated salt ions, such as Na^+^ (3.58 Å), Mg^2+^ (4.28 Å), and Ca^2+^ (4.12 Å).^132–134^ Experimental evidence shows that an interlayer spacing less than about 6.5–7.0 Å is sufficient for the effective exclusion of these ions based on size. It has also been shown that the interlayer spacing can be intentionally widened by inserting other metal ions (such as Al^3+^ and Fe^3+^) or organic molecules (such as DMSO and urea) to increase the water permeability.^135^ However, the balance between permeability and selectivity is optimal within about 10 Å, indicating the need for optimizing the ion rejection and permeability balance.^136^ It has been found that several stabilization methods can be used for overcoming the problem of interlayer swelling in an aqueous environment, such as cross-linking agents (*e.g.*, glutaraldehyde and polyethylenimine) and pillaring methods.^137,138^ The work of Lu *et al.*^139^ found that self-cross-linked MXene membranes (SCMM) produced by –OH condensation with –O bridges between the layers were able to suppress swelling effectively (Fig. 8a). The *d*-spacing of a dry pristine MXene membrane (PMM) was found to be 13.6 Å, while that of the SCMM was slightly smaller at 12.8 Å. More significantly, when PMM was exposed to water, it expanded significantly to 16.6 Å while SCMM expanded only slightly to 15.4 Å. The results show that cross-linking is an effective method to stabilize the interlayer spacing. Similarly, Long *et al.*^140^ showed that it is possible to control the interlayer spacing by adjusting the ratio of MXenes to Al_2_O_3_ in the membrane assembly (Fig. 8b). With an increasing number of Al_2_O_3_ nanoparticles, the *d* spacing of the nanocrystals in the dry-state increased steadily up to 1.56 nm at a ratio of 1 : 3 MXene : Al_2_O_3_. This proved the successful intercalation of nanoparticles between layers of MXenes. The resulting wide and stable nanochannels not only gave an improvement in the properties of the membrane surface but also in the overall transport performance. Size-selective transport is governed by a geometric parameter (interlayer spacing), while surface chemistry is the chemical parameter that determines the interactions between the walls of the nanochannels and water and ions. Thus, knowledge of surface chemistry is also crucial for the prediction of membrane performance.

The surface chemistry and hydrophilicity of MXene membranes are the key factors to determine their water purification and contaminant removal performance. The hydroxyl (–OH), oxygen (–O), and fluorine (–F) functional groups on the surface of MXenes determine the interaction between water and the membrane, as well as the management of ions and foulants during filtration.^141^ One of the main conclusions that can be drawn from the literature is that the surface of MXenes is significantly more water-attractive when it is terminated with –OH and –O. The water contact angle of MXene membranes is typically between 20° and 40°, while for other two-dimensional membranes like graphene and MoS_2_, it is generally greater than 60°.^142–144^ This high hydrophilicity enables the quick formation of a hydration layer, and consequently a high-water flux and stable long-term operation. Another key factor is the surface charge that develops when MXene membranes come into contact with water. It is a result of –O and –OH groups and causes electrostatic repulsion of the negative species. Hence, the membranes exhibit both Donnan exclusion of anions and antifouling properties.^145^ The pH-responsive surface properties represent an additional functionality of MXenes. Alkaline conditions lead to deprotonation of –OH groups and consequently to a higher negative surface charge, resulting in reduced fouling and higher rejection of contaminants. Protonation in an acidic environment reduces the surface charge, and in specific separation applications and reactive membrane design, this can be utilized.^146–148^ Molecular dynamics simulations performed by Ma *et al.* were helpful in providing mechanistic insights. They found that the contaminant removal performance depends on both surface termination type and interlayer spacing. The strength of water-channel interactions was found to be OH > O > F, with fluorine-terminated membranes having the highest water permeability, and OH-terminated membranes having the lowest (Fig. 8c). All the terminations, however, were able to successfully remove Na^+^ ions because of the negative surface charge. In order to achieve the optimal balance between water flow and contaminant rejection, an interlayer spacing of the Ti_3_C_2_F_2_ membrane of 9 Å was used (Fig. 8d).^144^ Experimental research by Meng *et al.* also showed that surface chemistry is important. They functionalized self-crosslinked MXene membranes with polyethyleneimine (PEI), thereby changing the surface charge from negative to positive in a range of pH of 4.5–9.0. This modification not only improved the hydrophilicity but also increased the electrostatic repulsion to cations; the transport rate of ions decreased by up to two orders of magnitude, the swelling of the MXene was avoided, and the size-sieving function was maintained. This led to an increase in MgCl_2_ rejection from 35% to 82% while sustaining the water permeance (Fig. 8e).^119^ Beyond size exclusion, the collective evidence from simulations and experiments indicates that ion rejection arises from a coupled mechanism. This mechanism includes partial dehydration of ions, electrostatic repulsion from charged surfaces, and the specific arrangement of water molecules along the hydrophilic basal planes of MXene layers.^149,150^ Whether the transport property is maintained during long-term operation or not depends on the mechanical stability, while surface chemistry dictates the selective transport. Thus, mechanical robustness is also a key requirement, and if it is inadequate, then even the best interlayer spacing and surface chemistry will not withstand real-world use.

The structural stability and mechanical strength of MXene membranes are crucial for their water purification application.^151^ The inherent properties of MXenes, such as Ti_3_C_2_T_*x*_, are flexibility and strength because of the 2D architecture with strong in-plane metallic bonding,^152^ but the tendency of individual nanosheets to restack during fabrication is another important challenge that can lead to reduced interlayer spacing, loss of water permeability, and mechanical fragility.^153^ To overcome this, several reinforcement methods have been developed and tested successfully such as cross-linking with polymers (PVA, chitosan, polyethyleneimine, *etc.*) and intercalation with nanomaterials (such as graphene oxide, CNTs, and MOFs) that have proved to be highly effective at improving the integrity of the membrane structure.^154,155^ Mechanical stability is also enhanced by advanced fabrication methods, including layer-by-layer assembly, vacuum-assisted filtration and hot-pressing, which help to ensure well-aligned laminar structures, with strong interlayer hydrogen bonding and van der Waals forces that prevent delamination under shear stress.^156^ Moreover, MXene membranes also exhibit outstanding chemical and thermal resistance, maintaining structural stability at temperatures of up to 800 °C in an inert atmosphere and stability in the pH range of 3–10.^157,158^ In conclusion, pristine MXenes were theoretically found to be mechanically (Young's modulus: 92 161 N m^−1^) and dynamically (stiffness up to 300 Nm^−1^ with terminations of O and F) stable, as confirmed by the DFT calculations,^159^ carried out by Yorulmaz *et al.* Experimental studies by Chen *et al.* showed that the electrical conductivity (10 400 S cm^−1^), tensile strength (112 MPa), and strain energy at failure (1480 kJ m^−3^) of Ti_3_C_2_T_*x*_ films were significantly enhanced, due to the enhancement of interlayer interactions and the absence of weakly bonded species.^160^ Likewise, Zhang *et al.* prepared a flexible porous silicon/MXene composite with outstanding mechanical resilience with a sheet-like porous structure and structural cavities that could accommodate up to 300% volume expansion (Fig. 8f–j).^161^ Together, these results validate the possibility of enhancing the mechanical stability of MXene membranes *via* appropriate material fabrication techniques, cross-linking strategies, nanomaterial intercalation, and surface termination engineering, thereby ensuring the long-term durability and performance of the membranes for their intended applications, such as desalination and water treatment.

## 3 Types of MXene-based membranes

MXene-based membranes have attracted attention due to their unique properties and characteristics. Their capacity to enhance both flux and selectivity has led to their potential as next-generation separation membranes.^154^ Different types of MXene-based membranes along with their performance matrices are summarized in Table 2.

**Table 2:** Comparative analysis of various parameters of different types of MXene-based membranes

Membrane type/composition	Modification	Key mechanism or feature	Performance/results	References
Pristine MXene (Ti_3_C_2_T_*x*_)	None (pure laminar film)	Hydrophilic surface; charge & size selective transport	High ion selectivity; permeability; swelling reduces performance	107 and 154
MXene–CA composite (CCAM-10%)	MXene in cellulose acetate	Hydrophilic channels	99.5% BSA; 96.6% dye rejection	178
MXene–PSF composite	Phase inversion embedding	Strong bonding; hydrophilicity	Flux 49%; FRR 76.1%	179
MXene–PVDF hollow fiber	Chemical grafting	Hydrophobic–hydrophilic interface	156.2° contact angle; stable flux	180
MXene–CNT hybrid	CNT spacing	Prevents restacking; redox active	202× permeability; 99.8% Au(iii) rejection	169
MXene–GO composite	Heat crosslinking	GO–MXene covalent bonding	Na^+^ rejection 99.3%	170
Ag-NPs–GO/MXene	Ag-NP coating	Antibacterial; antifouling	99% oil rejection; FRR 96.9%	171
MXene-BIC@MIL-101(Fe)	Self-assembly	Photocatalytic self-cleaning	Flux 2534 L m^−2^ h^−1^; 99.7% separation	172
MXene–ZnO hybrid	ZnO intercalation	Superhydrophilic; photocatalytic	Flux 1815–2053; 99.4% separation	173
Cross-linked MXene	Thermal OH–OH link	Ti–O–Ti bonding	Improved stability; no swelling	181
Metal-ion cross-linked	Al^3+^ insertion	Fixed nanochannels	95% spacing stability	175
Organic cross-linked	Phosphonic acids	Pillared structure	5× permeance	176
PIL–MXene composite	Poly(ionic liquid) intercalation	Tunable hydrophilicity	2× permeance; ∼99% rejection	177

### 3.1. Pure/pristine MXene membranes

**Fig. 9:**
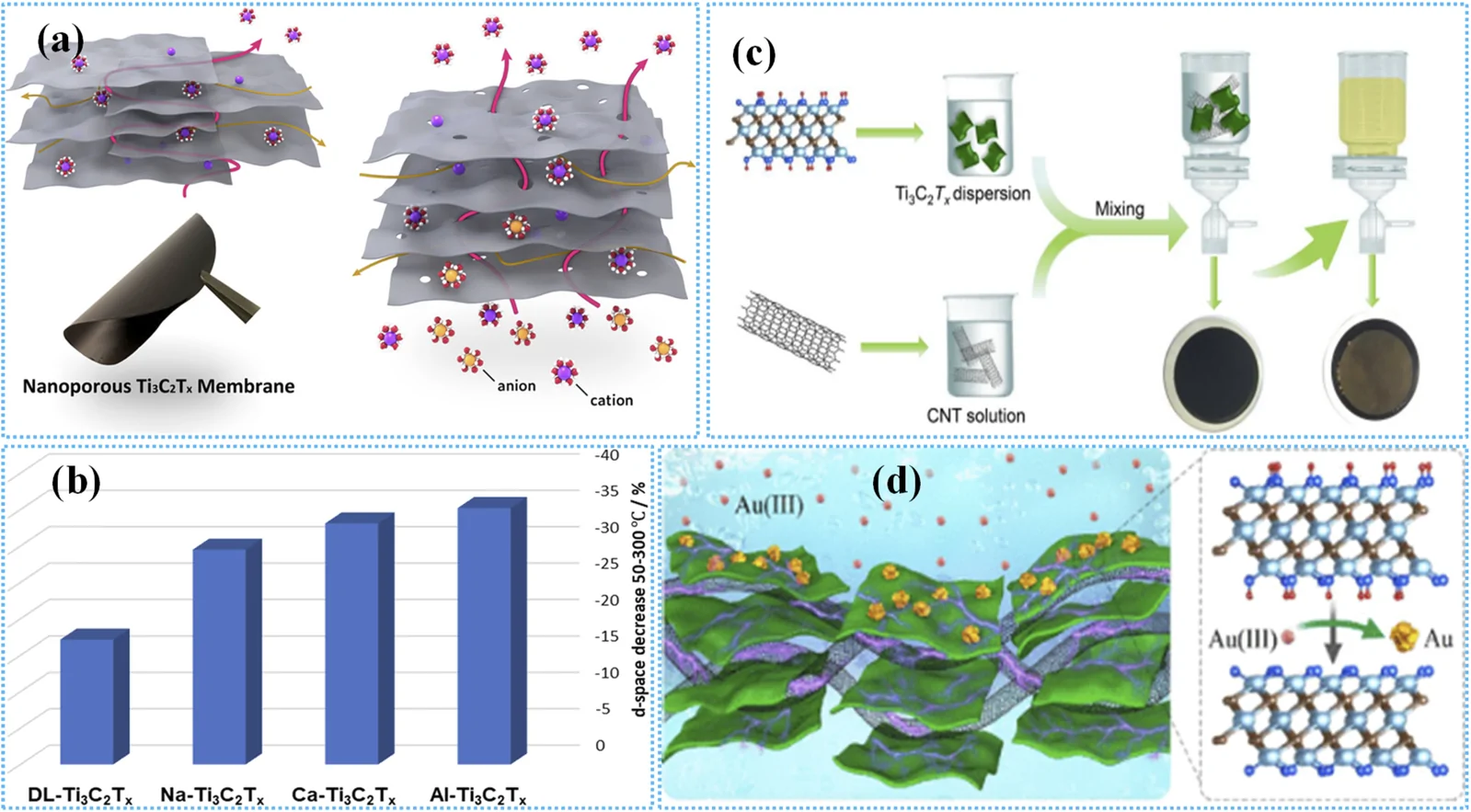
(a) Schematic of ion transport through the chemically etched nanopore and two-dimensional slit channels in lamellar MXene membranes. Reproduced from American Chemical Society from ref. 163, which is open access and permits unrestricted use of materials under the terms of the Creative Commons CC-BY. (b) *d*-Space (calculated from XRD analysis) variation between 50 and 300 °C. Reproduced from Royal Society of Chemistry from ref. 164, which is open access and permits unrestricted use of materials under the terms of the Creative Commons CC-BY. (c) Schematic illustration of the experiment process. (d) Schematic showing the redox reaction mechanism of Au(iii) rejection by the Ti_3_C_2_T_*x*_–CNT hybrid membrane. Reproduced with permission from ref. 169. Copyright 2020, American Chemical Society.

Pristine MXene membranes are generally a laminar film composed solely of two-dimensional transition metal carbide/nitride (*e.g.*, Ti_3_C_2_T_*x*_) nanosheets assembled into well-aligned nanochannels.^107^ Due to their hydrophilic nature and the presence of abundant surface functional groups, these membranes facilitate rapid water transport and effective molecular/ion sieving, while simultaneously filtering different substances based on size and charge.^36,162^ As a result, such membranes exhibit excellent ion and solute selectivity along with high water permeability, making them highly effective for water treatment applications such as desalination and ion-selective separation (Fig. 9a).^163^ In addition, pristine MXene membranes have demonstrated diverse water and ion transport behaviors, which highlight their great potential for developing advanced, high-performance separation applications. Furthermore, the hydrophilic and electrically conductive nature of pristine MXene membranes enables ultra-fast water flux and electro-assisted separation. Interestingly, MXene membranes offer charge and size selective separation, flexibility, tunable thickness, and high mechanical strength. Meanwhile, these membranes offer effective selective sieving of salts depending on their ion size and charge.^37^ Nevertheless, despite these benefits, the aqueous swelling effect of pristine MXene laminar membranes is still a major drawback. In particular, swelling results in the expansion of interlayer (*d*) spacing at the expense of mechanical stability and lowering ion sieving efficiency (Fig. 9b).^164^ To overcome these problems, several modification measures have been designed to correct the original flaws of pristine MXene membranes. The most recent of these are the MXene–polymer composite membranes, which represent an improved form of MMMs, in which two-dimensional MXene nanosheets are embedded in a polymer matrix. The method exploits the physicochemical characteristics of MXenes and the outstanding processability and mechanical strength of polymers.^11,12^ MXene nanosheets in polymeric membranes (polyvinyl alcohol (PVA), cellulose acetate (CA), or polyethersulfone (PES)) exhibit higher water permeability, antifouling behavior, and selectivity than pure polymer membranes.

### 3.2. MXene nanoparticle hybrid membranes

To enhance the quality of MXene membranes, various materials are added as alteration strategies to obtain high ion rejection and high permeation at the same time, including carbon nanotubes, graphene oxides, metal–organic frameworks, covalent organic frameworks, metal-coated polymers, *etc.*.^165^ In general, the functionalization of MXene membranes with a variety of nanoparticles, such as GO, MOFs, COFs, and Fe_3_O_4_, is a flexible and promising approach for the modification of the physicochemical characteristics of the membranes. These hybrid membranes have shown better separation factors, antifouling, and structural stability and therefore are good prospects in the application of high-water purification and sustainable water production processes.^166–168^

**Fig. 10:**
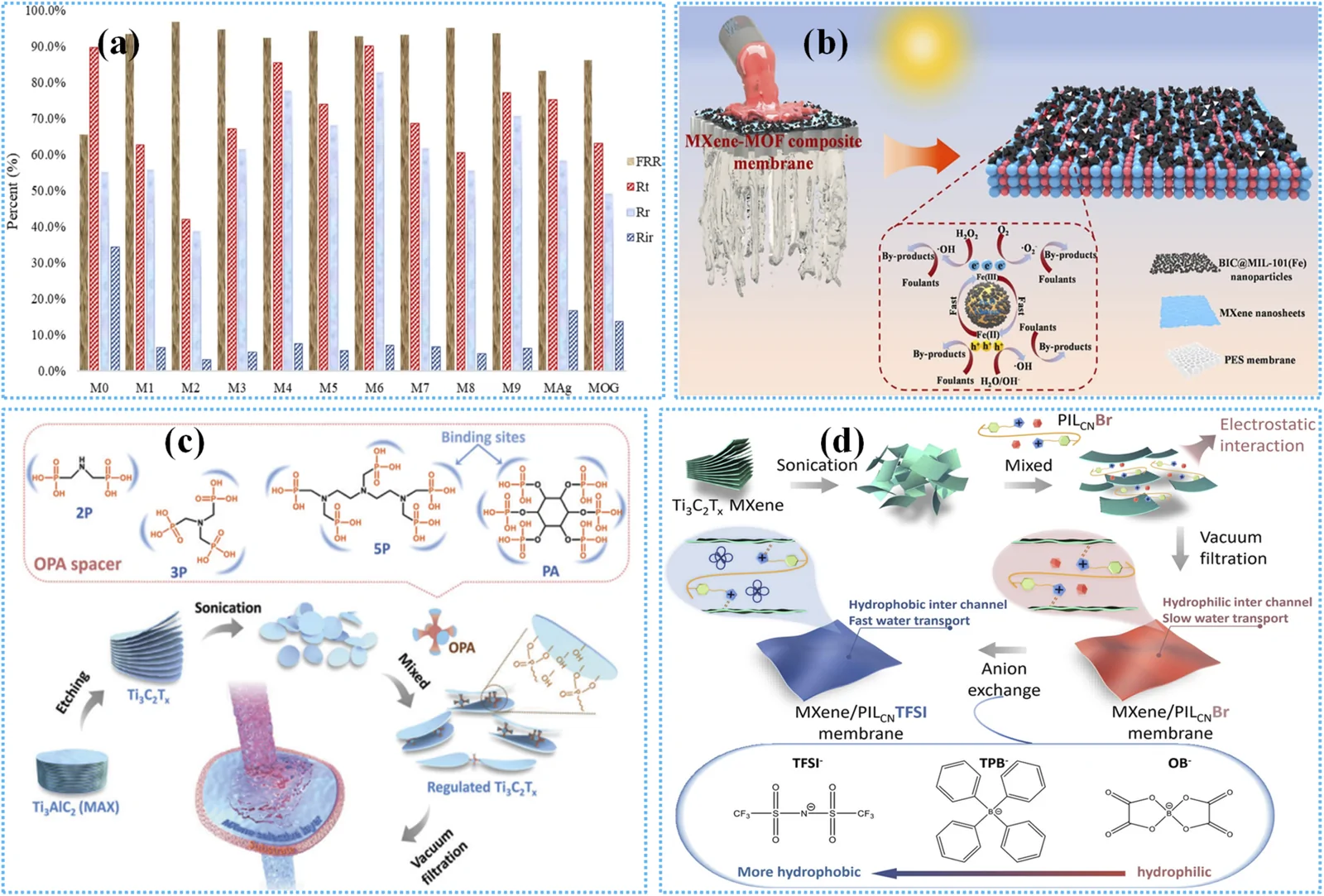
(a) Membrane permeability FRR, Rir, Rr, and Rt of the different fabricated membranes Reproduced from Springer Nature from ref. 171, which is open access and permits unrestricted use of materials under the terms of the Creative Commons CC-BY. (b) Schematic illustration of the self-cleaning mechanism in MXene–MOF membranes for oil–water separation. Reproduced with permission from ref. 172. Copyright 2025, Elsevier. (c) Schematic of the fabrication of organic phosphonic acid (OPA) modified MXene membranes. Reproduced from Royal Society of Chemistry from ref. 176, which is open access and permits unrestricted use of materials under the terms of the Creative Commons CC-BY. (d) Schematic of the fabrication process of MXene/PIL membranes. Reproduced from Wiley from ref. 177, which is open access and permits unrestricted use of materials under the terms of the Creative Commons CC-BY.

For instance, a sandwiched Ti_3_C_2_T_*x*_–CNT hybrid membrane was developed, in which CNTs prevent MXene restacking, enhance structural stability, and act as spacers for fast water transport (Fig. 9c). As a result, the membrane showed a permeability of 437.6 L m^−2^ h^−1^ bar^−1^, which is nearly 202 times higher than that of pristine Ti_3_C_2_T_*x*_, while simultaneously achieving 99.8% Au(iii) rejection even at 20 ppm (Fig. 9d). This outstanding performance arises from the redox activity of surface C–Ti–OH groups, which enables selective metal reduction. Therefore, Ti_3_C_2_T_*x*_–CNT membranes provide a sustainable platform for precious metal recovery from wastewater.^169^ In another study, through heat-induced bonding, GO carboxyl groups were chemically linked with MXene OH groups, thereby stabilizing the interlayer spacing, which consequently, reduced ion permeation rates by 7–40 times compared to pristine membranes and achieved 99.3% Na^+^ rejection (as compared to 80.8% in pristine).^170^ Similarly, AgNP-coated GO/MXene nanocomposite membranes were created to tackle wastewater treatment and membrane fouling. In this system, the membranes showed high mechanical strength, hydrophilicity, and antibacterial activity, achieving >99% oil rejection with a flux of 160 L m^−2^ h^−1^ and FRR of 96.9% (in comparison to 65.6% for neat PA6) (Fig. 10a). Furthermore, zeta potential and Hermia model analysis confirmed improved anti-fouling behavior and bacterial inactivation against *E. coli* and *S. aureus*.^171^ Complementing this, MXene-BIC@MIL-101 (Fe) composite membranes with photocatalytic self-cleaning properties were fabricated *via* self-assembly (Fig. 10b). The catechol-modified MIL-101(Fe) enhanced hydrophilicity, light absorption, and antifouling behavior, thereby enabling fluxes of 2534 L m^−2^ h^−1^ bar^−1^ with 99.7% oil–water separation efficiency. Importantly, the membranes maintained 90% flux recovery after 10 cycles, demonstrating long-term operational stability. Thus, the MXene–MOF synergy offers a sustainable route for anti-fouling and high-performance oily wastewater treatment.^172^ Similarly, MXene–ZnO hybrid membranes, fabricated *via* tannic acid-assisted intercalation of ZnO nanoparticles into MXene nanosheets, overcome swelling and restacking by effectively enlarging interlayer spacing, creating superhydrophilic/underwater superoleophobic surfaces (WCA = 0°, UOCA >152°). As a result, they exhibit high flux (1815–2053 L m^−2^ h^−1^), >99.4% oil/water separation efficiency, ultra-low oil adhesion, and strong antifouling performance with photocatalytic self-cleaning (methylene blue degradation) and antibacterial activity. In this hybrid system, ZnO provides structural stability, photocatalysis, and microbial resistance, while MXenes ensure lamellar channels and durability under salty and high-temperature conditions.^173^

### 3.3. Cross-linked or intercalated MXene membranes

A cross-linked MXene membrane is proposed to be one of the best modified forms of MXene membranes for suppressed swelling properties, where nanosheets are bound through hydrogen bonding, covalent bonding, and ionic interaction, taking advantage of the hydroxyl terminal groups on the MXene nanosheets, for example, by forming Ti–O–Ti bonds between neighboring nanosheets *via* self-crosslinking through a facile thermal treatment.^109,122^ Covalent crosslinking has been demonstrated as a valid approach to impose restrictions on the interlayer spacing of hydrophilic laminate membranes. Cross-linkable functional group or monomer incorporation into the membrane structure may lead to the formation of covalent bonds between the layers, enhancing stability and leading to a controlled interlayer spacing.^158^ Additionally, for stabilizing the MXene laminar membrane, metal ion modification can be used. For example, for the angstrom-scale interlayer spacing in Ti_3_C_2_T_*x*_, Al^3+^ insertion *via* molten-salt vacuum hot-pressing is used even under dynamic stress, conforming robust anti-swelling performance and retaining >95% spacing stability after 500 cycles.^174^ As a complementary strategy, alginate hydrogel pillars cross-linked by multivalent cations are utilized, which fix the nanochannel size at 7.4 ± 0.2 Å and deliver outstanding separation performance, including complete Na_2_SO_4_ rejection, 80% NaCl rejection, high water permeance (28 L m^−2^ h^−1^ bar^−1^), and superior H^+^/Fe^2+^ selectivity.^175^

Organic molecules have emerged as powerful modifiers for engineering the interlayer galleries of MXene membranes, enabling precise control over channel size, hydrophilicity, and long-term stability. For instance, a series of organic phosphonic acids (OPAs) was employed as multifunctional spacers to enlarge and stabilize the interlayer spacing of Ti_3_C_2_T_*x*_ laminates (Fig. 10c). Through the formation of covalent Ti–O–P bonds between MXene flakes and phosphonic acid groups, pillared structures with well-defined nano-channels were achieved, resulting in a five-fold enhancement in water permeance while retaining high rejection and remarkable tolerance in aqueous environments.^176^ In parallel, poly (ionic liquids) (PILs) have been introduced as organic intercalants to construct lamellar MXene membranes with tunable hydrophilicity. By chaining the counter anions within poly ionic liquids (PILs), the interlayer nano-channels were converted from hydrophilic to hydrophobic states, reducing water-wall friction and allowing the flow of water more easily while maintaining 99% rejection (Fig. 10d). These strategies together highlight the versatility of organic modifiers in pillaring, crosslinking, and hydrophilicity regulation, offering synergistic improvements in permeation, selectivity, and stability, and thus positioning organic–MXene hybrids as a more advanced frontier in water purification methodologies.^177^

## 4 MXene-based membranes in water purification

### 4.1. Desalination

Desalination refers to the chemical process of purifying salty water to make it drinkable by eliminating excessive salt and dissolved minerals. With the increased scarcity of water in the world, desalination has gained more significance in municipal, industrial, and commercial fields. MXenes and their membrane technologies have received considerable interest in this respect due to their high hydrophilicity, high surface area, and good mechanical and electrical qualities, which make them very appropriate in the development of sophisticated water purification technologies.^182^ Based on these characteristics, MXenes have been popular in a variety of desalination processes, such as adsorption, membrane separation, and capacitive deionization (CDI).^53,183,184^ Their chemical versatility allows size and charge selective rejection of ions and molecules, particularly when the nanosheets of MXenes are stacked into a freestanding or supported membrane. An example is Ti_3_C_2_T_*x*_ MXene, which has one of the lowest contact angles of 21.5°, which implies that it has a high affinity to water. It is also very stable even at a high rate of shaking, and this also reinforces its applicability in desalination systems.^185^ The layers of MXenes can be stacked together, which enables the separation of the molecules by size exclusion as well as by the electrostatic forces to the negatively charged surfaces.

**Fig. 11:**
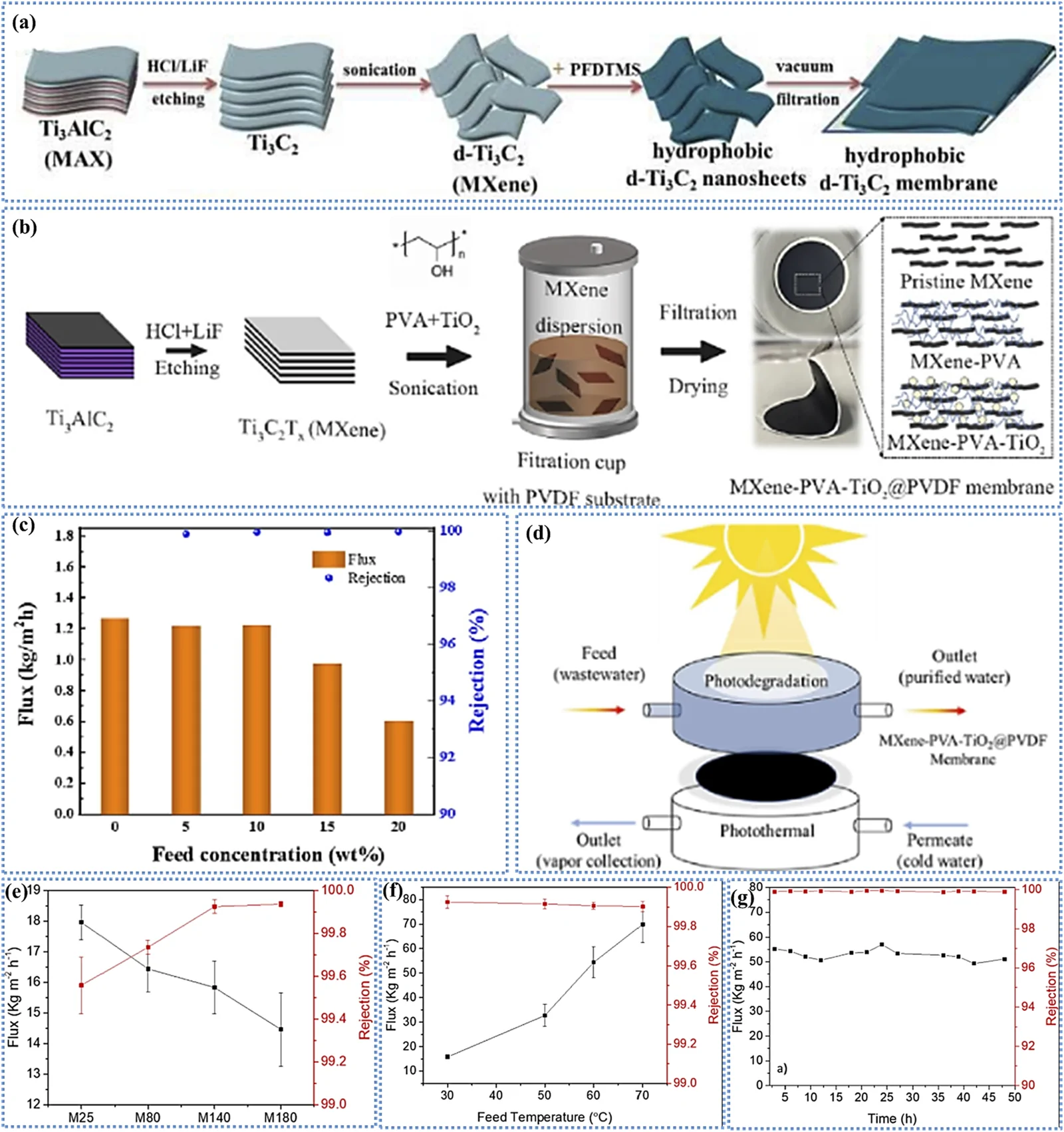
(a) Schematic illustration of the fabrication process of the hydrophobic *d*-Ti_3_C_2_ membrane. Reproduced with permission from ref. 186. Copyright 2018, Royal Society of Chemistry. (b) Schematic illustration of MXene preparation and MXene–PVA–TiO_2_ membrane fabrication, (c) the PMD performance of the MXene–PVA–TiO_2_@PVDF membrane at different feed concentrations, and (d) the schematic of the photothermal-catalytic membrane distillation system. Reproduced with permission from ref. 187. Copyright 2023, Elsevier. (e) PV performance test results of M25, M80, M140, and M180 membranes at a feed concentration of 10 wt% NaCl and feed temperature of 30 °C, (f) effect of feed temperatures for 10 wt% NaCl feed solution with the M140 membrane, and (g) long-term PV test results using simulated brine and the M140 membrane at 70 °C feed temperature for 48 h. Reproduced with permission from ref. 188. Copyright 2023, Elsevier.

The other potential avenue of MXene materials is pervaporation-based desalination. This is done by the diffusion of water through a membrane and then evaporation on the permeate side. To promote this strategy, Zhao *et al.* prepared a hydrophobic MXene membrane in the form of a delaminated Ti_3_C_2_ layer that had been treated with trimethoxy (1H, 1H, 2H, 2H-perfluorodecyl) silane (Fig. 11a). Their integration of the MXene layer with a commercial filter membrane produced a self-floating device that was able to collect solar power effectively. This device achieved a high solar evaporation rate of 1.31 kg m^−1^ h^−1^ under one-sun radiation and demonstrated excellent operational stability for more than 200 h under high salinity conditions due to the rapid water-transport capacity and efficient salt-blocking behavior.^186^ Sun *et al.*, in a similar development, made a stable MXene–PVA–TiO_2_@PVDF Janus membrane to be used in a photo thermal-catalytic membrane distillation (PMD) system (Fig. 11b). The resultant membrane was found to have improved desalination operation with a flux of 1.23 kg m^−2^ h^−1^ and 99.9% salt rejection (Fig. 11c). This work further demonstrates how modifying MXene surfaces can significantly elevate their photothermal efficiency in desalination applications (Fig. 11d).^187^ Further innovations in MXene-based pervaporation membranes were demonstrated by Fareed *et al.*, who optimized an additive-free self-crosslinking process to fabricate MXene-coated membranes. Prepared through vacuum filtration followed by controlled thermal crosslinking, the membrane cross-linked at 140 °C (M140) exhibited the best performance. It maintained stable interlayer spacing in both pure water and salt solutions and delivered a permeation flux of 70 kg m^−2^ h^−1^ with 99.9% salt rejection at 70 °C (Fig. 11e and f). Impressively, the membrane maintained 50 kg m^−2^ h^−1^ flux and complete salt rejection during a 48 h continuous operation test, confirming its long-term stability (Fig. 11g).^188^Table 3 summarizes the previously reported MXene-based membranes for desalination performance.

**Table 3:** MXene-based membrane performance in desalination of water

Membrane	Interlayer space	Thickness (µm)	Test duration (min)	Contact angle (°)	Water flux (kg m^−2^ h^−1^)	Water permeability (L m^−2^ h^−1^ bar^−1^)	Salt	Rejection (%)	References
PFDTMS modified *d*-Ti_3_C_2_ membrane	0.137–1.507 nm	2	12 000	102	1.31	—	CuCl_2_, K_2_Cr_2_O_7_	71	186
MXene–PVA–TiO_2_@PVDF					1.23	—	NaCl	99	187
MXene membrane M140	1.34–1.48 nm	3	48	—	50–70	—	NaCl	99.90	188
COOH–MXene/PA	3.6 to 4.0 Å	1	12 000		—	12.9	MgSO_4_	99.1	189
							NaCl	11.4	
Ti_3_C_2_T_*x*_/PVPA composite	—	0.5	420	50–100	—	0.2	Na_2_SO_4_	>97	190
							K_2_SO_4_	80	
MoS_2_@MXene composite	1.38–1.52 nm	0.2	—	77.2–48	—	172.5	Na_2_SO_4_	82.5	191
							MgSO_4_	75	
							NaCl	60	
							MgCl_2_	50.58	
PLA/TiO_2_nfs/MXene fibrous	—	0.35	60	—	1.0	—	Sea water	99.95	192
MXene–TFC NF	—	0.020–0.065	3000	43.6	—	45.7	Na_2_SO_4_	>96	193
Ultrathin MXene membrane	—	∼8	6000	45.9	—	85.4	NaCl	99	194
CAM	—	340	360	—	1.47	—	NaCl	92.1	195
MXene/PA-2 NF	1.37–1.43 nm	0.0581–0.0989	4800	70	—	20.4	Na_2_SO_4_	97.3	196
CD/TMC polyester-interlayers of Ti_3_C_2_T_*x*_ MXene membranes	174–427 Å	0.1509–0.2403	1440	88.30	—	93–149.06	MgSO_4_	93.96	197
							Na_2_SO_4_	93.00	
							MgCl_2_	85.88	
							NaCl	76.25	
MWCNT–Ti_3_C_2_ MXene	—	6.9–7.7	4	49.2–25.7	1.55	—	Na^+^, K^+^, Mg^2+^and Ca^2+^ salts	>99.5	198

### 4.2. Heavy metals

The hydrophilicity of MXenes, combined with the large quantity of active functional groups present on their surfaces, allows them to be an effective adsorbent of a large variety of ionic and molecular pollutants.^199^ They have many chemically and electrostatically active sites on their surfaces and hence, have a high affinity with metal ions and are therefore efficiently captured in their treatment processes. The most dangerous pollutants of soil and water include heavy metals, such Cu^2+^, Cr^6+^, Cd^2+^, Pb^2+^, and Hg^2+^, which are highly toxic, and hence their elimination is of utmost importance.^200^ In this regard, incorporation of MXene nanosheets into membrane scaffolds has become an attractive approach to embrace adsorption and selective separation processes to remove heavy metals. The membranes based on MXenes take advantage of their hydrophilicity, adjustable interlayer spacing, as well as charged surface properties to produce high permeation fluxes and outstanding ion rejection and adsorption properties.^37,107^ Stacked MXene membrane interlayer nanochannels with size sieving ability, and the large surface terminations of the membranes, including –OH and –O, promote electrostatic interactions and surface complexation, which allow easy size-based elimination and chemical capture of heavy metal species.^201^ In addition, MXene composite membranes in the presence of polymers, nanoparticles, or other two-dimensional materials (*e.g.*, GO, CNTs, and Fe_3_O_4_) have been demonstrated to be highly beneficial to mechanical stability, fouling resistance, and separation performance.^202^ As an example, functionalized MXene membranes have shown rejection efficiencies exceeding 95–99% of different toxic metal ions such as Pb^2+^, Ni^2+^, Cd^2+^, and Cu^2+^ under optimal conditions with a high water permeability.^203^ It is also possible to control membrane architecture by surface modification or intercalation to control channel size and surface charge, which allows selectivity in the removal of targeted heavy metals even when using complex aqueous conditions.^204^

**Fig. 12:**
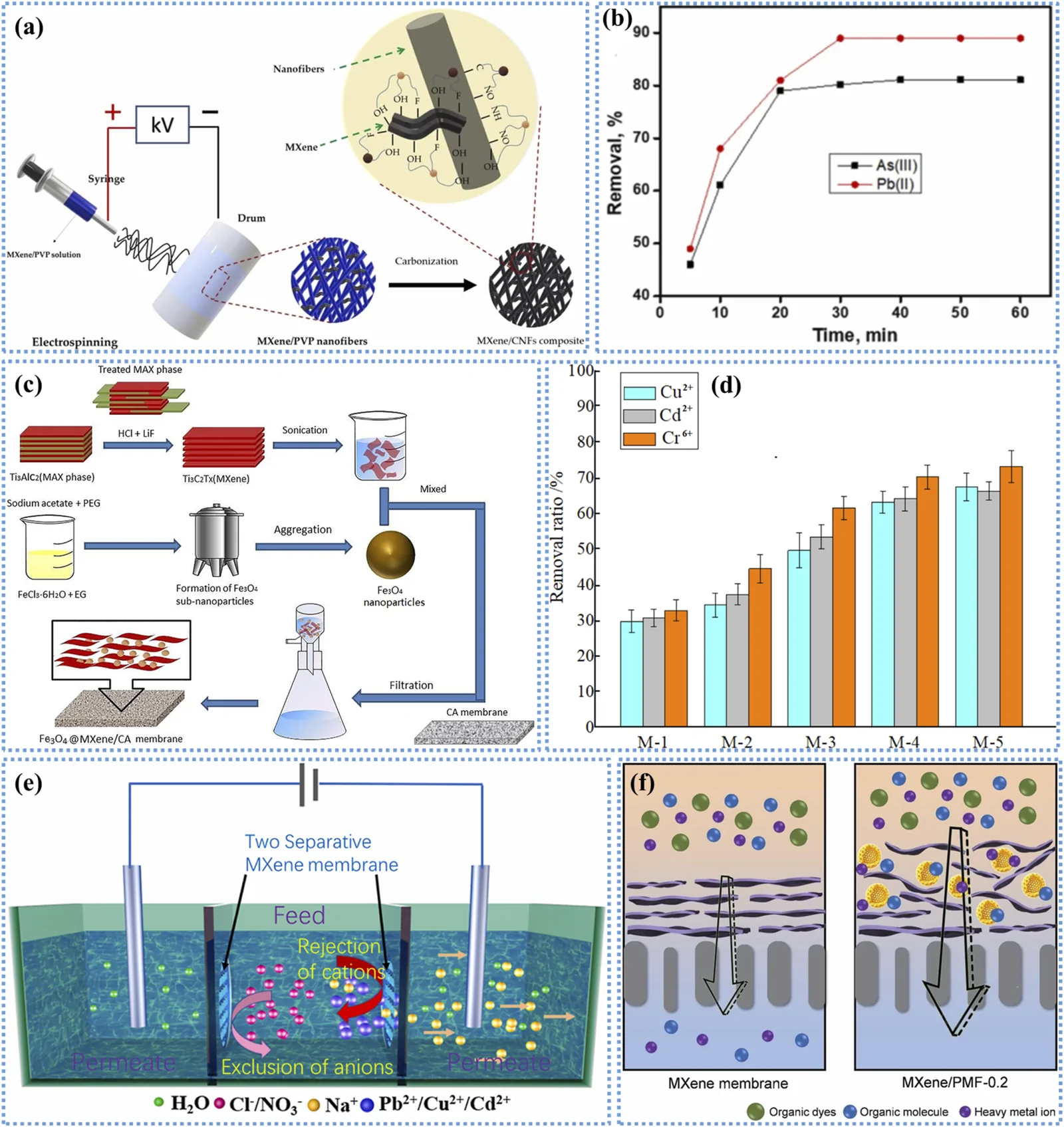
(a) Graphical representation of carbonization and electrospinning of the MXene/CNF nanofiber membrane preparation; (b) time effect on adsorption of Pb^2+^ and As^3+^ using MXene/CNFs. Reproduced with permission from ref. 205. Copyright 2023, Elsevier. (c) Schematic illustration of the construction of the Fe_3_O_4_@MXene/CA composite NF membrane; (d) removal ratio of heavy metal ions by different composite NF membranes. Reproduced with permission from ref. 206. Copyright 2020, Wiley. (e) Graphical illustration of hydroxylation of MXene membranes and their removal pattern. Reproduced with permission from ref. 207. Copyright 2021, Elsevier. (f) The ability of the MXene/PMF composite membrane to remove heavy metal ions and its water permeability of 381.2 L m^−2^ h^−1^ bar^−1^. Reproduced with permission from ref. 208. Copyright 2024, Elsevier.

For instance, Mahar *et al.*, in this regard, designed MXene/carbon nanofiber (MXene/CNF) composite nanofiber membranes that were able to remove Pb^2+^ and As^3+^ ions to acceptable levels (Fig. 12a). Under neutral pH conditions, the composite membrane showed adsorption capacities of Pb^2+^ (12.5 mg g^−1^) and As^3+^ (3.3 mg g^−1^), which had a removal efficiency of 89 and 81%, respectively, in 20–30 min (Fig. 12b). Furthermore, the membrane demonstrated strong recyclability, maintaining removal efficiencies of 77% for Pb^2+^ and 60% for As^3+^ after 4 regeneration cycles.^205^ Similarly, Yang *et al.* fabricated Fe_3_O_4_@MXene nanocomposite membranes by self-assembling Fe_3_O_4_ nanoparticles and MXene nanosheets onto cellulose acetate substrates (Fig. 12c). Owing to the synergistic effects of nanolayered sieving and enhanced surface adsorption, the resulting nanofiltration membrane achieved removal efficiencies of 63.2% for Cu^2+^, 64.1% for Cd^2+^, and 70.2% for Cr^6+^ (Fig. 12d).^206^ Building on structural modification strategies, Wang *et al.* demonstrated that hydroxylation of MXene membranes achieved through substituting surface –F groups with –OH using KOH significantly enhances membrane wettability and surface charge (Fig. 12e). These improvements substantially increase rejection performance. Under an applied electric field, the 383 nm-thick hydroxylated membrane achieved exceptional rejection of heavy-metal cations (Pb^2+^, Cu^2+^, and Cd^2+^) at 99.5%, while simultaneously excluding coexisting anions (Cl^−^ and NO_3_^−^) at approximately 97%. Furthermore, it is seen that the membrane had unique selective permeability to Na^+^, in contrast to Pb^2+^, Cu^2+^, and Cd^2+^.^207^ In addition, Zhang *et al.* created an MXene/poly-melamine-formaldehyde (PMF) composite membrane where PMF particles serve as nanoscale spacers (Fig. 12f). The combination of the synergistic interaction between the –NH_2_ of PMF and the –OH of MXene, and the crosslinking of glutaraldehyde to ensure that the membrane was intact, led to significantly improved performance. The composite with 83.7% PMF had a water permeability of 381.2 L m^−2^ h^−1^ bar^−1^ that was more than four times higher than that of pristine MXene membranes and high removal efficiencies of Zn^2+^ (96.2%) and Pb^2+^ (91.7%), which were significantly higher than the results of unmodified MXene membranes.^208^Table 4 presents a summary of previously reported MXene-based membranes for heavy metal removal.

**Table 4:** Summary of MXene-based membranes for heavy metal removal

Membrane	Interlayer space	Thickness (µm)	Test time (min)	Water permeability (L m^−2^ h^−1^ bar^−1^)	Water flux (L m^−2^ h^−1^)	Heavy metal	Removal (%)	References
MXene/CNFs	1.29–6.89 nm	—	20	—	—	Pb^2+^	89	205
						As^3+^	81	
Fe_3_O_4_@MXene	—	0.0034	60		125.1	Cu^2+^	63.2	206
						Cd^2+^	64.1	
						Cr^3+^	70.2	
Hydroxylated MXene	15.08 Å	0.383	70	—	—	Pb^2+^, Cu^2+^,Cd^2+^	99.5	207
MXene/PMF	—	7	—	381.2	—	Zn^2+^	96.2	208
						Pb^2+^	91.7	
MXene/GO/chitosan (CMG)	4.7 Å	10	480		36.7–42.8	Co^2+^	78	137
						Cu^2+^	76	
Cross-linked MXene (Ti_3_C_2_T_*x*_)	6.7–6.92 Å	0.365	60	—	—	Pb^2+^	99	209
Ti_3_C_2_T_*x*_–EDA	—	—	—	21.1	—	Mn^2+^, Zn^2+^, Cd^2+^, Cu^2+^, Ni^2+^, Pb^2+^	93–99.8	210
PEI-modified GO/MXene	14.7 Å	0.275	300	9.8	—	Ca^2+^, Mg^2+^	70	211
MM8S–COOH membrane	1.54–1.77 nm	0.020	60	1.97–3.08	—	Hg^2+^	99.7	212
Ti_3_C_2_T_*x*_/mycelium hybrid	—	10	60	51 800	—	Pb^2+^	87–99	213
MXene-modified PVC mixed matrix	—	200	—	279.81	—	Pb^2+^	96.86	214
ST@PA composite NF (STPP)	—	—	10 080	24.6	—	Mn^2+^	98.8	215
						Ni^2+^	95.3	
						Cu^2+^	95.7	
						Zn^2+^	97.3	
Alk-MXene/Co-MOF	—	10.2–18.8	400	443.7	—	Cr^6+^	98	216
Multi-layer MXene 1%	—	—	2880	121.6	—	Pb^2+^	73.7	217

### 4.3. Oil–water separation

Oily wastewater has been released due to oil spills and other industrial activities, thus causing serious threats to humanity and global ecosystems. Thus, it is necessary to create high-efficiency separation membranes because membrane-based technologies are still one of the most effective methods of treating oil wastewater.^218^ It is important to note that MXene membranes are characterized by excellent long-term stability and antifouling properties when compared to traditional membrane materials. Furthermore, these membranes exhibit increased rejection of oil when salt-containing oily wastewater is present, which indicates the distinctive characteristics of MXene 2D lamellar architecture.^219^

**Fig. 13:**
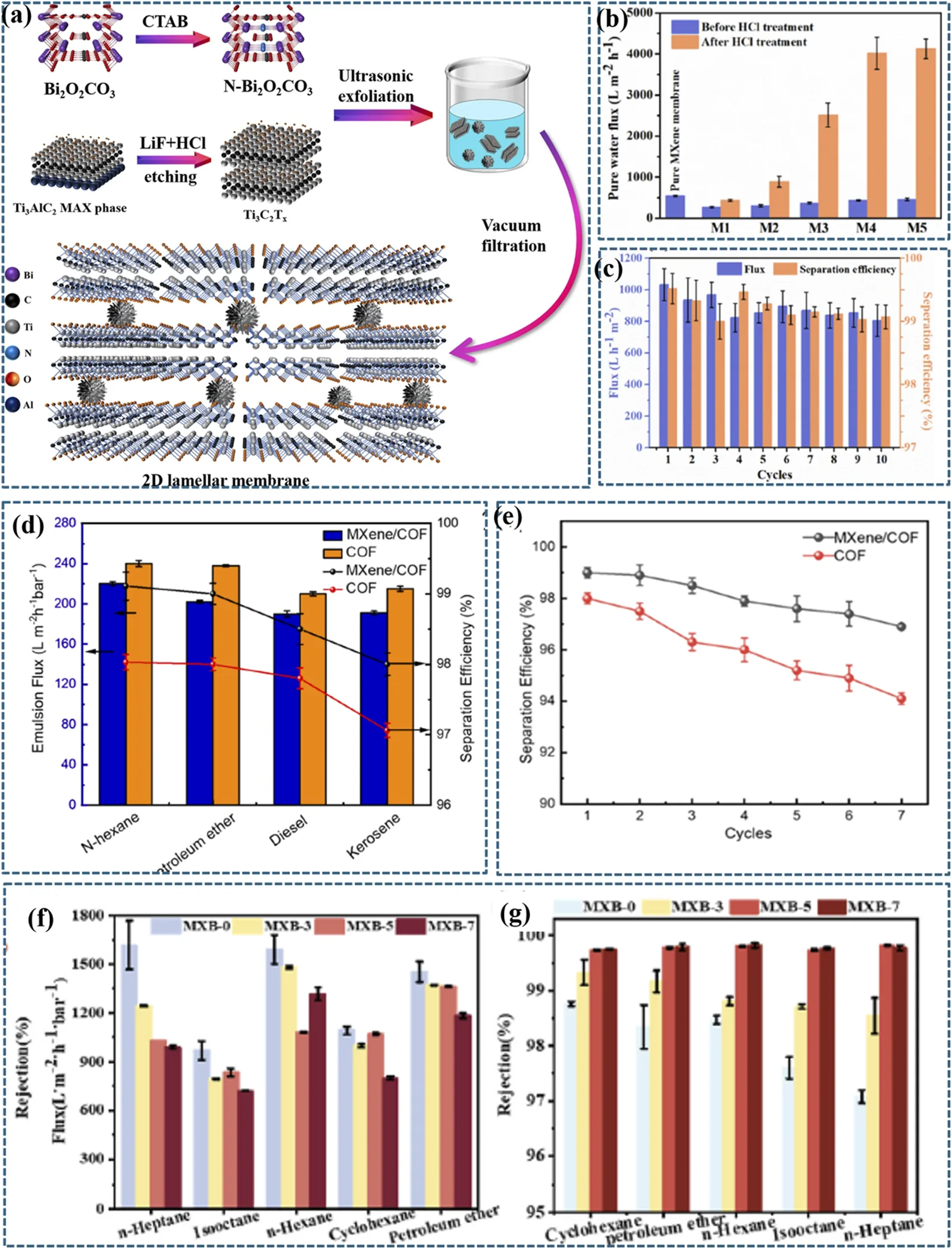
(a) Schematic illustration of the construction process of the N–Bi_2_O_2_CO_3_@MXene/PES composite membrane. Reproduced with permission from ref. 220. Copyright 2022, Elsevier. (b) Pure water flux of the pristine MXene membrane and a series of MXene membranes prepared by using different volume ratios of Fe (OH)_3_ colloidal solution to Ti_3_C_2_T_*x*_ MXene solution before and after HCl treatment under 0.08 MPa and (c) the flux and separation efficiency of MCT-FeOOH10 membranes separating an isooctane-in water emulsion during cyclic experiments. Reproduced with permission from ref. 221. Copyright 2021, Elsevier. (d) Emulsion flux and separation efficiency of am-3D-S-COF and MXene/am-3D-S-COF membrane for various oil-in-water emulsions. (e) Separation efficiency of oil-in-water emulsions (petroleum ether) for seven cycles. Reproduced with permission from ref. 222. Copyright 2025, Elsevier. (f and g) Emulsion flux and rejection of MXB-*X* membranes (*X* = 0, 3, 5, 7) for various oil–water emulsions. Reproduced with permission from ref. 172. Copyright 2025, Elsevier.

In this direction, Lin *et al.*^220^ synthesized 2D Bi_2_O_2_CO_3_/MXene photocatalytic composite membranes with versatile water-treatment functions (Fig. 13a). N-doped Bi_2_O_2_CO_3_ nanoparticle application considerably enhanced the performance of the used membrane with a very high-water flux of 815.3 L m^−2^ h^−1^ and oil/water emulsion rejection at a rate of over 99%. Similarly, Hu *et al.* developed a super-hydrophilic and underwater super-oleophilic MXene-based composite membrane by vacuum-assisted self-assembly of MXene nanosheets onto a porous PVDF substrate, followed by *in situ* mineralization of the β-FeOOH photo catalyst. The prepared membrane achieved high permeation fluxes ranging from 500.38 to 1022.7 L m^−2^ h^−1^ and excellent separation efficiencies of 99.32–99.72% for various micro-scaled oil-in-water emulsions (Fig. 13b and c). These improvements were attributed to the membrane's inherent super-wettability and its controlled lamellar structure, which provides short transport pathways and numerous nanochannels.^221^ Furthermore, Qu *et al.* designed a super-wettable MXene/3D-S-COF composite membrane by depositing MXene nanosheets onto an oxidized amide COF layer formed from an imine COF. The hydroxyl groups of the COF not only enhanced water permeability but also formed hydrogen bonds with the MXene layer, producing a robust and highly stable composite architecture. As a result, the synergistic interplay between the porous 3D-S-COF network and the zigzag MXene arrangement delivered an emulsion flux of 240 L m^−2^ h^−1^ bar^−1^ and a separation efficiency exceeding 98% (Fig. 13d and e).^222^ Similarly, Li *et al.* reported photocatalytic self-cleaning MXene–MOF composite membranes fabricated through self-assembly. These membranes exhibited outstanding performance, achieving a flux of 2534.89 ± 13.63 L m^−2^ h^−1^ bar^−1^ and a separation efficiency of 99.7% (Fig. 13f and g). The water contact angle decreased to below 5° within just 5 seconds, attributed to the hydroquinone-functionalized coating on the MIL-101(Fe) surface. This coating effectively minimized oil adhesion and imparted excellent self-cleaning characteristics to the membrane.^172^Table 5 depicts the reported literature of MXene-based membranes for oil-efficient oil–water separation.

**Table 5:** Performance of MXene-based membranes in oil water separation

Membrane	Interlayer spacing	Thickness (µm)	Reusability (cycles)	Contact angle (°)	Water permeability (L m^−2^ h^−1^ bar^−1)^	Water flux (L m^−2^ h^−1)^	Oil type	Efficiency (%)	Reference
Bi_2_O_2_CO_3_@MXene	17.0 Å	28–30	5	71.2–50	—	815.3 L m^−2^ h^−1^	Vegetable oil	99.6	220
							Lubricant oil	98.8	
2D MXene@β-FeOOH	—	—	10	160	—	500.38–1022.7	Oil-in-water emulsions	99.32–99.72	221
MXene/3D-S-COF	—	0.0031	—	35–0	240	—	Oil-in-water emulsions	>98	222
MXene-BIC@MIL-101(Fe) composite	—	0.0002–0.001	10	5	2534.89	—	Oil-in-water emulsions	99.7	172
Ti_3_C_2_T_*x*_ on PVDF	—	18.15 l	10	158	887	—	Kerosene	>99.4	223
PES–MXene@TiO_2_ composite	14.46–15.87 Å	0.225–0.355	10	158	3045	—	*n*-Hexane, cyclohexane, *n*-heptane, isooctane, petroleum ether, and gasoline	>99.8	224
Co-PA/SL-Ti_3_C_2_T_*x*_	—	—	—	130–5	9200–22 100	—	Water/vegetable oil emulsions	>99	225
TA-ZIF-8@MXene/CA	—	12	8	149.2–153.1	5347.36	—	Vegetable and lubricant oil	>98	226
Cracked-earth-like hydroxyl-rich Ti_3_C_2_T_*x*_	1.45, and 0.95 nm	—	5	157	6385	—	Toluene, petroleum, ether, kerosene, and *n*-hexane	>72 (mg L^−1^)	227
Hal@MXene–PDA two-dimensional (2D) composite	13.4–17.6 Å	20–50	3	70–22.1	5036.2	—	Petroleum ether and lubricating oil	99.8	228
MXene	—	1.2	8	137	>450	—	Diesel, silicone oil, petroleum ether, and hexane	>99	229
Sep@MXene/CA composite	—	1.97–9.99	3	18.2–69.2	611.2–820.3	—	Lubricating oil emulsion	>95	230
FM@MXene	—	—	10	74.5	—	3.75 × 10^3^	*n*-hexane and edible oil	99	231
APTES/MXene–ACNTs/CA	—	—	9	77.1–55.0	2892.8		Lubricating oil emulsion and vegetable oil emulsion	>99	232
MXene@COF-TpBD-PES composite	0.33 nm	0.5	20	150	1908.4		Oil–water emulsion	99.78	233
Sulfonated polydopamine functionalized MXene (SPDMX)	9.875 A	1000	15	153	1620		Surfactant-stabilized oil-in-water emulsion	98	234

### 4.4. Dye and pharmaceutical removal

Synthetic dyes and pharmaceutical compounds have been of great concern to the global environment due to their persistence, toxicity, and high mobility in water bodies since the pollutants may build up in the environment and eventually cause a negative impact on plants, animals, and human lives.^220,235,236^ These bioactive pharmaceutical residues, which are not always degraded and thus enter natural water systems, constitute a significant ecological threat because of their bioactivity and the promise to interfere with the functioning of natural ecosystems, but MXene-based materials,^236,237^ especially when used as catalytic platforms, have shown an outstanding capacity to stimulate the degradation rate of such recalcitrant compounds due to their special physico-chemical properties and high surface reactivity.^238,239^ Considering these characteristics, the recent literature has heavily highlighted the fact that MXene-derived nanomaterials have great potential in terms of adsorption of dyes and various pharmaceutical residues in aqueous systems.^141^

**Fig. 14:**
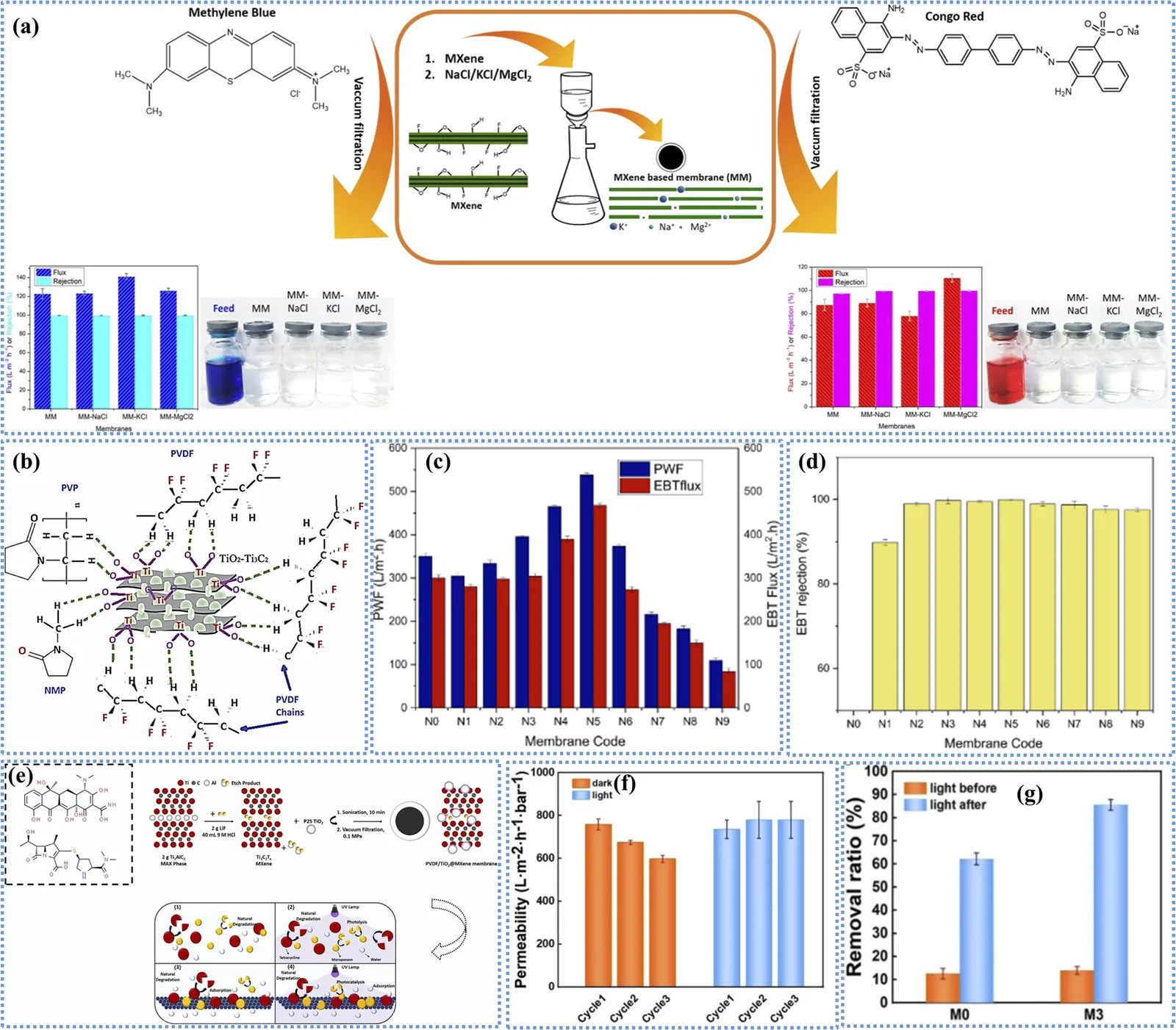
(a) Schematic illustration and CR and MB separation by the chloride salt modified MXene membrane. Reproduced with permission from ref. 240. Copyright 2022, Elsevier. (b) The proposed interaction mechanism among components of the prepared membrane, (c) pure water and EBT flux of membranes and (d) Eriochrome black T rejection of the neat PVDF and MXene modified membranes. Reproduced from Elsevier from ref. 241, which is open access and permits unrestricted use of materials under the terms of Creative Commons CC-BY. (e) Schematic illustration of the fabrication of PVDF/MXene@TiO_2_ membranes. Reproduced with permission from ref. 242. Copyright 2024, Elsevier. (f) The permeability and removal ratios and (g) TC removal ratios of M0 and M3. Reproduced with permission from ref. 243. Copyright 2022, Elsevier.

One such modification strategy that could have a powerful impact, reported by Sagita *et al.* is the addition of chloride salts (NaCl, KCl, and MgCl_2_) into MXene membranes, a process that significantly enhanced their separating capabilities with both cationic dyes (*e.g.*, methylene blue, or MB) and anionic dyes (*e.g.*, Congo red) (Fig. 14a). The KCl-modified MXene (MM-KCl) was found to have the highest flux (141 L m^−2^ h^−1^) and total rejection of MB, which the authors explained by the weaker hydration of the water molecules by potassium ions, which allowed faster movement of water. On the other hand, for CR filtration, MM-MgCl_2_ exhibited the highest performance, with a flux of 117 L m^−1^ h ^−1^ and nearly 100% rejection, demonstrating that the MXene-based materials can be tuned for ion-specific interactions.^240^ Furthermore, by MXene-enabled membrane engineering, Abood *et al.* prepared an asymmetric ultrafiltration membrane by adding MXenes to a PVDF base material through bulk modification, which greatly enhanced the antifouling resistance of the membrane and stability of its functioning during textile wastewater treatment (Fig. 14b). The resulting nanocomposite membranes demonstrated a remarkably high pure-water flux of 538 LMH and retained a remarkable 467.8 LMH of Eriochrome Black T (EBT) dye solution, and the optimized membrane composition had almost perfect rejection of EBT (Fig. 14c). Furthermore, the flux recovery ratio showed a steady increasing tendency, finally peaking at 85.6% when membranes were prepared using 0.5 wt% MXene, indicating the strong antifouling properties of the membrane (Fig. 14d).^241^ The same trend of improvement was also observed in the work of Kadadou and Hasan, who produced a two-dimensional PVDF/TiO_2_@MXene composite membrane to eliminate antibiotics, namely, tetracycline and meropenem (Fig. 14e). During the introduction of TiO_2_ nanoparticles into the MXene matrix, the hydrophilicity and water permeation of the membrane were significantly enhanced, as indicated by a significantly low water contact angle of 19.5° and a permeation rate of 293 L m^−2^ h^−1^ bar^−1^. The composite membrane displayed good antibiotic separation properties with a tetracycline and meropenem rejection rate of 87.8% and 60.7%, respectively, which is substantiation that TiO_2_ and MXene contribute synergistically to greater pollutant selectivity.^242^ Moreover, Zeng *et al.* presented a new process of wastewater treatment by altering MXene membranes with g-C_3_N_4_ (CN) nanosheets to produce a new photocatalytic g-C_3_N_4_@MXene/PES (CN-MX) composite membrane with the method of vacuum filtration. This composite membrane was able to show very high permeability (1790 L m^−2^ h^−1^ bar^−1^) due to the optimization of the layered structure and synergistic photocatalytic characteristics (Fig. 14f). Significantly, the CN-MX membrane showed excellent removal capacity towards a variety of organic pollutants with 86% removal of tetracycline hydrochloride, having a high rejection rate of 80%, illuminating the prospect of photocatalytically active MXene composites in the development of advanced water purification methods (Fig. 14g).^243^Table 6 provides a summary of various MXene-based membranes reported for dye and pharmaceutical pollutant removal.

**Table 6:** MXene-based membrane performance for dye and pharmaceutical removal

Membrane	Modification	Interlayer space	Thickness (µm)	Time (min)	Contact angle (°)	Water permeability (L m^−2^ h^−1^ bar^−1^)	Water flux (L m^−2^ h^−1^)	Pollutants	Efficiency (%)	Reference
MXene	KCl and MgCl_2_	—	3 to 4	—	<60	—	141	Methylene blue	100	240
						—	117	Congo red		
MXene–PVDF	Polyvinylidene fluoride	—	—	90	85.5	467.8		Eriochrome black T	85.6	241
PVDF/TiO_2_@MXene	Multi-layer TiO and PVDF		16	180	19.5	293	—	Tetracycline	87.8	242
								Meropenem	60.7	
g-C_3_N_4_@MXene/PES (CN-MX)	g-C_3_N_4_	15.7–16.2 Å	1.67–29	480	71.76	1790	—	Congo red	98	243
								Trypan blue	96	
								Tetracycline hydrochloride	86	
CMG catalytic nanocomposite	MXene, graphene oxide, and chitosan	—	—	480	80–85	—	42.8	Methylene blue	96	244
MXene/Ti	2D ultrathin	—	—	12	—	—	—	Tetracycline hydrochloride	90.4–100	245
Ti_3_C_2_T_*x*_–MXene	Ascorbic acid and aluminum ion	1.49 Å	1	40–60	—	0–1200	—	dyes	99.33	246
BiOCl-PPy@MXene	BiOCl-PPy	—	10.2–18.8	400	—	3680.2	—	Congo red and trypan blue	99.9	247
							—	Tetracycline hydrochloride	96	
Bi_2_O_2_CO_3_@MXene	N-doped Bi_2_O_2_CO_3_ nanoparticles	13.8–17.0	1.5–30	480	71.2	—	815.3	Congo red	99.9	220
								Trypan blue	98	
								Rhodamine B	98.4	
Silane-grafted MXene	*n*-Octadecyl trichlorosilane (MODCS), *n*-octyl trichlorosilane (MNOCS), and triphenyl chlorosilane (MTPCS)	—	—	—	—	—	—	Methylene blue and Sudan II	52–98	248
Cyclodextrin-decorated MXene	Supramolecular cyclodextrins	1.40–1.42 nm	30.3	105	35.0	431.37	—	Methylene blue	>99.7	249
Ti_3_C_2_T_*x*_ MXene-coated	Poly(ether sulfone)	14.3 nm	—	—	58.9–11.5	2420	—	Amitriptyline	50.9	250
						2440		Ibuprofen	36.2	
GO-Ti_3_C_2_T_*x*_	Lamellar GO	8.8–15.2 Å	2	—	54.5–61.6		83.8	Tetracycline	99.8	251

### 4.5. Antimicrobial and antifouling properties

Biofouling is one of the biggest issues in membrane-based separation mechanisms and is associated with the deposition of bacterial cells and their growth upon the surface of the membrane, which ultimately results in the development of a thick biological layer that drastically reduces the membrane structure, selectivity, and life span. The antifouling properties of Ti_3_C_2_T_*x*_ MXene are also enhanced by its intrinsic antibacterial effect, which affects or impairs the integrity of the cell membranes and subsequently leads to structural disruption and subsequent cell death.^252^ Besides this contact-based antibacterial effect, the high hydrophilicity and negative charges of Ti_3_C_2_T_*x*_ MXene membranes have a vital effect on reducing the rate of bacterial adhesion and organic matter deposition, and therefore inhibiting the initial phases of biofilm formation.^47^ The existence of surface terminations like –OH and –O will encourage the creation of a hydration layer on the membrane surface that will serve as a mechanical barrier to microbial attachment and deposition of extracellular polymeric substances (EPSs).^180,253,254^ Moreover, MXene composite membranes have also shown enhanced resistance to biofouling over a long period of operation and stable separation behavior, which suggests that these membrane types can be used to extend the operational lifetime of water treatment systems.^255^ Irrespective of these benefits, the long-term stability of MXene membranes under realistic operating conditions, as well as the understanding of the antifouling mechanism, is also another significant research area to implement them in practice.^256^ In addition, particular biochemical elements of the microbial cell walls and the cytoplasm may react with Ti_3_C_2_T_*x*_, further weakening cellular structures and making bacteria die faster, and the naturally reduced size of different microorganisms makes MXene-assembled membranes especially effective in selective biological separation.^158^

**Fig. 15:**
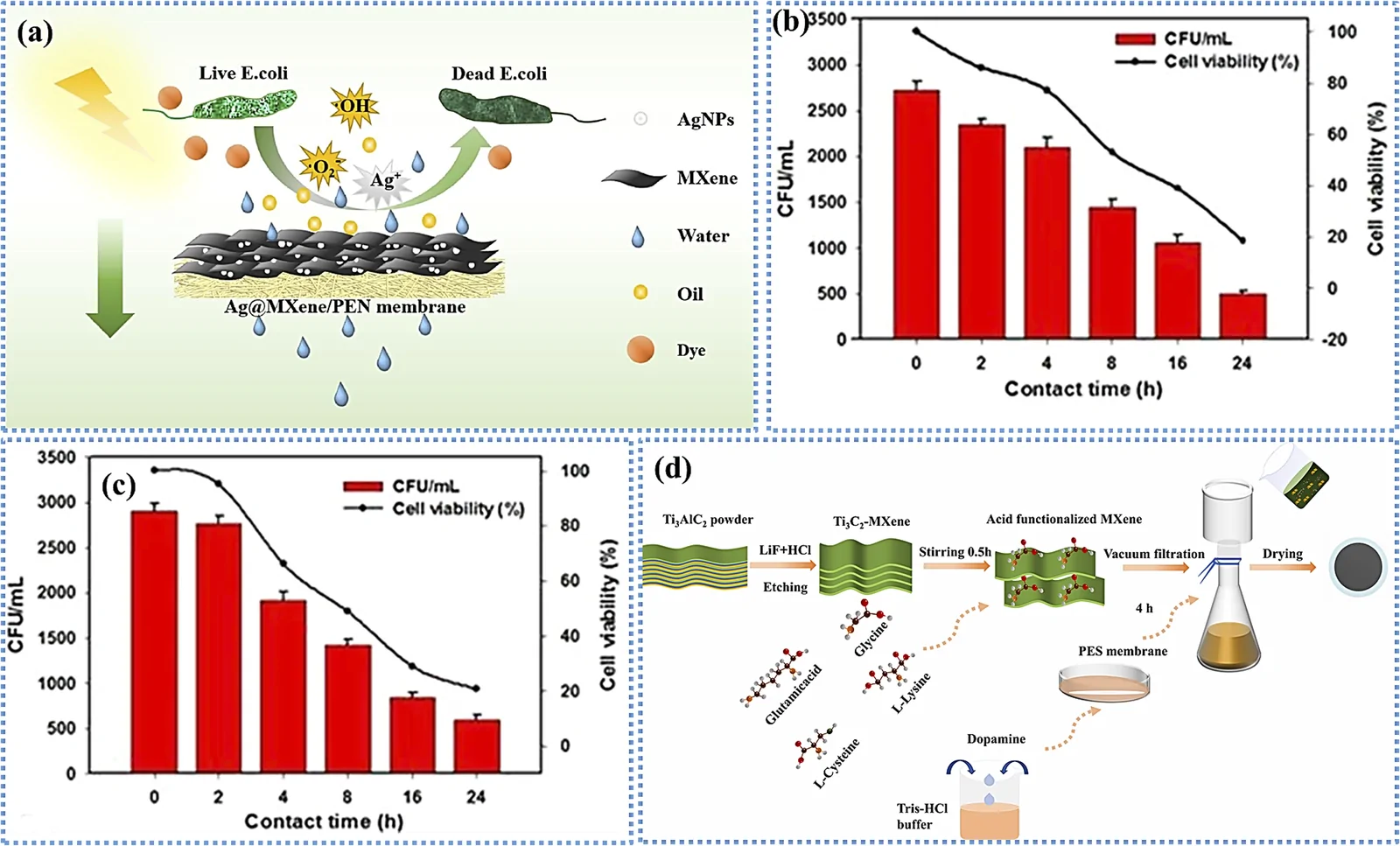
(a) Separation and anti-fouling mechanism of the Ag@MXene/PEN fibrous composite membrane. Reproduced with permission from ref. 257. Copyright 2022, Elsevier. (b) Cell viability measurements of *E. coli* and (c) *B. subtilis* exposed to MXene membranes at different time intervals during 24 h of contact time. Reproduced from Elsevier from ref. 47, which is open access and permits unrestricted use of materials under the terms of the Creative Commons CC-BY. (d) Schematic illustration of the amino acid-bonded Ti_3_C_2_T_*x*_ MXene NF membrane preparation. Reproduced with permission from ref. 49. Copyright 2024, Elsevier.

In the given description, Feng *et al.* created a multifunctional Ag@MXene/PEN fiber composite membrane that was manufactured by the self-assembly of Ag@MXene hybrids onto an electrospun poly(arylene ether nitrile) (PEN) support, which led to a high antibacterial efficiency of the membrane, reaching 99.99% growth inhibition against *E. coli* (Fig. 15a).^257^ Correspondingly, Rasool *et al.* reported that Ti_3_C_2_T_*x*_ MXene membranes on polyvinylidene fluoride (PVDF) supports exhibited high antibacterial activity with fresh membranes showing 73% to 67% against *B. subtilis* and *E. coli*, respectively, and aged Ti_3_C_2_T_*x*_ membranes showing even higher activity, 99% against both strains; flow cytometry also verified that about 70% of bacterial cells were dead or damaged 24 h later (Fig. 15b–c).^47^ Furthermore, for enhancing the structural and functional versatility of MXene-based membranes, Luo *et al.* reported a family of amino-acid-modified Ti_3_C_2_T_*x*_ membranes functionalized with glycine (Gly), l-glutamic acid (L-Glu), l-lysine (L-Lys), and l-cysteine (L-Cys). Among these, the Gly-bonded membrane had the best nanofiltration properties (Fig. 15d). It provides high salt rejection and superior antifouling characteristics, with a flux recovery rate of 80.59% against bovine serum albumin (BSA).^49^ Addressing the persistent challenge of low flux and membrane fouling in separation processes, Pandey *et al.* proposed Ag-decorated MXene (Ag@MXene) membranes as a promising solution, demonstrating that the 21% Ag@MXene composite membrane not only achieved excellent organic molecule rejection and good flux recovery but also provided robust antibacterial protection, inhibiting over 99% of *E. coli* growth, in contrast to the 60% inhibition observed with pristine MXene membranes relative to the control PVDF membrane.^117^Table 7 summarizes the previously reported MXene-based membranes for antimicrobial performances along with different metrics.

**Table 7:** Summary of MXene-based composite membranes for antibacterial performance

Membrane	Targeted bacteria	Antibacterial performance (%)	Antifouling properties	References
Ag@MXene/PEN	*E. coli*	99.99	Super hydrophilic and photocatalytic self-cleaning	257
Ti_3_C_2_T_*x*_ MXene/PVDF	*E. coli*	67	Bacterial cell surface damage	47
	*B. Subtilis*	73		
Amino acid-bonded Ti_3_C_2_T_*x*_			Gly-bonded: FRR = 80.59% (BSA fouling)	49
Ag@MXene	*E. coli*	> 99	High rejection of BSA and methyl green	117
Ti_3_C_2_T_*x*_ MXene@Ag nanowire	*E. coli* and *S. aureus*	>99	High BSA rejection (∼95.4%)	258
Ti_3_C_2_T_*x*_-coated PVDF	*E. coli*	—	Prevented biofilm formation; antibiofouling on the membrane surface	259
MXene-coated cellulose	Broad antibacterial test	>99.9	Stable for long-term wastewater treatment	182
PES composite membranes with Nb_2_AlC and Mo_3_AlC_2_ MAX phases	*E. coli*, *S. aureus*, and *P. aeruginosa*	100, 69.74	Enhanced antifouling against BSA and microbial biofilms	260
Ti_3_C_2_T_*x*_/CNF-14	*Escherichia coli*	99.89	Multifunctional antifouling capability	261
TiO_2_/MXene composite ultrafiltration membrane	*Escherichia coli*	>95	—	262
PES/SPES-Ti_3_C_2_T_*x*_	*Bacillus subtilis*	—	>90% for Metanil yellow and methylene blue; salt rejection order: MgSO_4_ > Na_2_SO_4_ > NaCl	263
Ag@MXene/PHNF	—	99.8	Excellent resistance to biofouling	264
Au/Ti_3_C_2_	*Escherichia coli* K-12; *Sphingopyxis* sp. BM1-1	83.63	Good stability and resistance to biofouling during photo thermal evaporation	265
Ti_3_C_2_Tx@Cu-BTC@EP	*Escherichia coli*	100	Enhanced corrosion resistance	266
Nb_2_CT_*x*_Ag composite	*Staphylococcus aureus* and *Escherichia coli*	98.5	—	267

## 5 Conclusion

MXene-based membranes have rapidly emerged as one of the most promising classes of two-dimensional materials for advanced water purification technologies. Their unique physicochemical characteristics, including high electrical conductivity, tunable interlayer spacing, abundant surface functional groups (–O, –OH, and –F), and exceptional hydrophilicity, provide significant advantages over conventional polymeric membranes. These intrinsic properties enable efficient molecular sieving, strong electrostatic interactions with pollutants, and rapid water transport through well-defined nanochannels, thereby offering high permeance along with remarkable selectivity. This review comprehensively discussed the fundamental aspects governing MXene membrane technology, beginning with the synthesis strategies of MXenes derived from MAX phases. Conventional hydrofluoric acid etching, *in situ* fluoride generation methods, hydrothermal approaches, and emerging bottom-up routes such as the urea-glass method were examined in detail. Each synthesis pathway plays a critical role in determining the structural integrity, surface terminations, and interlayer characteristics of the resulting MXene nanosheets, which ultimately influence membrane fabrication and separation performance. Furthermore, various fabrication strategies for MXene membranes were analyzed, including free-standing lamellar membranes, mixed-matrix membranes, hybrid nanocomposite membranes, and cross-linked or intercalated architectures. Among these, hybrid and cross-linked MXene membranes have demonstrated superior structural stability and reduced swelling behavior, addressing one of the key limitations associated with pristine MXene laminates. The incorporation of secondary materials such as graphene oxide, carbon nanotubes, metal oxides, metal–organic frameworks, and polymers further enhances membrane permeability, antifouling behavior, antibacterial activity, and mechanical durability. These synergistic modifications allow the design of membranes with precisely engineered nanochannels and improved long-term operational stability. The application potential of MXene-based membranes in water purification was also critically reviewed. These membranes exhibit outstanding performance in a wide range of separation processes, including desalination, heavy-metal removal, oil–water separation, dye and pharmaceutical removal, and antimicrobial filtration. Their exceptional adsorption capacity, strong surface interactions with pollutants, and tunable charge-selective transport mechanisms enable high rejection efficiencies while maintaining significant water flux. As a result, MXene membranes are increasingly recognized as next-generation materials capable of addressing complex water contamination challenges. Overall, the integration of MXene nanostructures into membrane technology represents a transformative advancement in water treatment science. Continued progress in scalable synthesis, membrane engineering, and structural stabilization strategies is expected to further unlock the full potential of MXene-based membranes. With sustained research efforts and technological optimization, these materials hold great promise for developing high-efficiency, sustainable, and industrially viable solutions for global water purification and environmental remediation challenges.

## 6 Future perspectives

Despite the remarkable progress in MXene-based membranes for water purification, several scientific and technological challenges remain. Addressing these issues will be essential for translating laboratory-scale research into real-world applications. The following future research directions are particularly important.

### 6.1. Scalable and environmentally friendly MXene synthesis

Most MXenes are currently synthesized using hydrofluoric acid or *in situ* fluoride etching routes, which involve toxic reagents and generate hazardous waste. Developing greener and scalable synthesis methods remains a critical research priority. Future studies should focus on alternative etching strategies such as molten salts, electrochemical etching, alkali-assisted hydrothermal synthesis, and fluoride-free methods. The community should tackle the challenge of electrochemical delamination in non-corrosive electrolytes, such as NH_4_Cl or deep eutectic solvents based on choline chloride, that can also functionalize and exfoliate MXenes without producing corrosive waste. Besides, continuous flow reactor systems are all the more urgent to move from the gram-scale laboratory production to the kilogram per day industrial production with real-time quality control. These approaches could significantly reduce environmental hazards while improving large-scale production feasibility. Furthermore, continuous synthesis systems and industrial reactors may enable cost-effective manufacturing of MXene nanosheets for commercial membrane fabrication.

### 6.2. Long-term stability and oxidation resistance

One of the key limitations of MXene materials is their susceptibility to oxidation and structural degradation, especially in humid and oxygen-rich environments. Oxidation can lead to loss of conductivity, structural collapse of nanochannels, and deterioration of membrane performance. Future work should focus on stabilizing MXene structures through surface passivation, polymer encapsulation, and hybrid composite formation. A combination of hydrophilicity (required for water flux) and oxidation resistance (required by hydrophobic/passivated surfaces) presents a unique challenge in terms of core–shell architecture with a thin permeable surface layer that does not block the nanochannels. Furthermore, standardized accelerated ageing procedures need to be developed in the field to enable a comparison of membrane durability under a combination of factors (oxidants, UV, temperature cycling, and hydraulic shear). Encapsulation with graphene oxide, polymers, or metal oxides may effectively prevent oxidation and extend membrane lifespan under operational conditions.

### 6.3. Precise control of interlayer spacing

The separation performance of MXene membranes strongly depends on their interlayer nanochannel spacing, which governs molecular transport and ion selectivity. However, swelling in aqueous environments can cause uncontrolled expansion of these channels. Advanced strategies such as covalent crosslinking, organic molecule pillaring, polymer intercalation, and metal-ion bridging should be further explored to stabilize interlayer spacing. The key remaining challenge is to make the interlayer spacing dynamic and responsive to the stimuli, which can vary based on feed chemistry (*e.g.*, thin the interlayer for high salinity rejection and thickening it for high flux softening). Structure–performance relationships that are missed altogether in static, *ex situ* measurements could be uncovered by using machine learning and by performing *in situ* synchrotron XRD during the filtration process. Achieving angstrom-level control over nanochannels will enable highly selective separation of ions, pharmaceuticals, and emerging micropollutants.

### 6.4. Development of multifunctional hybrid membranes

Hybrid MXene membranes incorporating nanomaterials such as MOFs, COFs, CNTs, metal oxides, and photocatalysts offer promising pathways to combine multiple functionalities in a single membrane system. Future research should focus on designing membranes capable of simultaneous filtration, adsorption, photocatalysis, and antibacterial activity. The main limitation is not only increasing the number of components but also increasing the functionality synergically by the outer MXene layer (photocatalytic nanoparticles are often shadowed by this layer), which will be required to be addressed by future designs and using waveguide effects. Another possibility is self-regenerating membranes, which, having stored the chemical energy needed to break down foulants, would operate during idle times to a much greater extent, thereby dramatically lowering the frequency of chemical cleanings. Such multifunctional membranes could degrade organic pollutants while preventing fouling and microbial growth, thereby improving long-term performance in wastewater treatment systems.

### 6.5. Integration with advanced water treatment technologies

MXene membranes have shown great potential in several advanced processes such as capacitive deionization, membrane distillation, photocatalytic membrane reactors, and solar-driven desalination. Future research should explore the integration of MXene membranes with renewable energy systems and electrochemical treatment technologies. The most promising direction yet to be explored is the use of electrically conductive MXene membranes as self-cleaning electrodes, which can produce local reactive oxygen species directly on the surface by applying a low DC voltage (1–2 V) without requiring any separate chemical cleaning processes. This, however, can only be done if the current collectors, spacers, and seals of the small-scale lab cells need to be redesigned to avoid short circuits and uniform potential distribution in the spiral wound modules (m^2^).

### 6.6. Real wastewater testing and industrial implementation

Most studies on MXene membranes are conducted under controlled laboratory conditions using synthetic pollutant solutions. However, real wastewater contains complex mixtures of salts, organic compounds, microorganisms, and suspended particles. Future investigations should therefore emphasize long-term pilot-scale experiments using real industrial wastewater streams. One of the biggest challenges is the lack of long-term (≥6 months) pilot studies on real industrial or municipal wastewater, in which fouling due to natural organic matter and biofilms often leads to a 50–80% decrease in membrane flux within weeks, which does not often occur in synthetic wastewater pilot studies. Future work should not only be concerned with initial rejection, but also with flux decline kinetics, cleaning efficacy, and cost of m^3^ treated, as these are the metrics that are relevant for the end-user in industry and are vital for technology uptake. Such studies will provide valuable insights into membrane fouling behavior, durability, and economic feasibility under realistic operating conditions.

## Conflicts of interest

The authors declare that they have no conflict of interest.

## Data Availability

No new data were generated in this review.
